# The impact of social protection interventions on treatment and socioeconomic outcomes of tuberculosis-affected people and households in low income, high burden settings: A systematic review and meta-analysis

**DOI:** 10.1371/journal.pgph.0006704

**Published:** 2026-09-03

**Authors:** Mollie Hudson, Heather Todd, Talemwa Nalugwa, Ann Schraufnagel, Canice Christian, Delia Boccia, Tom Wingfield, Priya B. Shete

**Affiliations:** 1 Division of Pulmonary and Critical Care Medicine and Center for Tuberculosis, Zuckerberg San Francisco General Hospital, University of California San Francisco, San Francisco, California, United States of America; 2 Department of Social and Behavioral Sciences, School of Nursing, University of California San Francisco, San Francisco, California, United States of America; 3 Departments of Clinical Sciences and International Public Health, Liverpool School of Tropical Medicine, Liverpool, United Kingdom; 4 WALIMU, Kampala, Uganda; 5 Department of Epidemiology and Biostatistics, University of California, San Francisco, California, United States of America; 6 Department of Medical Sciences, Cancer Epidemiology Unit, University of Turin, Italy; 7 Centre for Tuberculosis Research, Departments of Clinical Sciences and International Public Health, Liverpool School of Tropical Medicine, Liverpool, United Kingdom; 8 Department of Global Public Health, Karolinska Institutet, Stockholm, Sweden; 9 Tropical and Infectious Diseases Unit, Liverpool University Hospitals NHS Foundation Trust, Liverpool, United Kingdom; University of Sydney, AUSTRALIA

## Abstract

Tuberculosis (TB) is the leading cause of death due to infectious disease worldwide. Social protection interventions can benefit TB-affected households. We conducted a systematic review and meta-analysis to quantify the effectiveness of social protection on TB treatment and socioeconomic outcomes. We identified articles published from January 2012 to December 2025 by searching PubMed (includes MEDLINE), Embase, and Web of Science. We included studies that described at least one social protection intervention and reported on either TB treatment or socioeconomic outcomes for people with TB or TB-affected households. Random-effects meta-analysis was used for our primary outcome of interest, TB treatment success (treatment completion or cure). We performed a meta-regression to evaluate the association of study characteristics with odds of TB treatment success. Risk of bias was assessed using the Newcastle Ottawa Scale and the Cochrane Risk of Bias tool. This review was registered prospectively in the PROSPERO database (registration number CRD42022382181). Our search generated 55,975 articles. Of the 55 which were eligible for inclusion, 41 reported TB treatment outcomes, 8 reported on socioeconomic outcomes, and two studies reported both TB treatment and socioeconomic outcomes. Random-effects meta-analysis of 26 articles found that people with TB who received social protection interventions during treatment had 2.15 times the odds of TB treatment success (95% CI 1.76, 2.64, I^2^ 94.5%). Subgroup analysis indicated that whether a social protection intervention was TB sensitive or TB specific was a significant source of heterogeneity, although both increased the odds of TB treatment success. Both TB sensitive and TB specific social protection interventions significantly improve odds of TB treatment success. Outcomes and definitions used in our study have the potential to guide further research and implementation of social protection for TB-affected populations.

## Introduction

Tuberculosis (TB) is one of the leading causes of infectious disease deaths worldwide. Despite effective and widely available treatment, an estimated 10.7 million people were infected with TB and 1.23 million people died from TB in 2024 [1]. TB-affected individuals are often trapped in a vicious cycle of poverty; impoverished individuals often have risk factors that make them more susceptible to TB (e.g., crowded living conditions, poor access to care, malnutrition), and becoming ill with TB often precipitates devastating economic consequences incurred from the such as costs associated with of care-seeking and treatment and lost wages due to missed work [2,3]. TB-affected households often use negative financial coping strategies and experience catastrophic costs, defined as total costs in excess of 20% of the annual household income [4].

To break out of the cycle of poverty and disease, interventions are urgently needed that address both underlying social and economic determinants and vulnerabilities of TB-affected individuals. Social protection interventions, broadly defined by the World Bank as systems that “help the poor and vulnerable cope with crisis and shocks, invest in the health and education of their children, and protect the aging population,” [5] are such a strategy. Social protection interventions are a key pillar of the World Health Organization (WHO) End TB Strategy, the United Nations’ Sustainable Development Goals (SDGs), and the UN Declaration on TB [6–8]. These interventions include cash transfers to eligible populations, job training programs to support income generation, and nutritional or food security programs. When implemented effectively, social protection can decrease TB incidence, improve TB treatment outcomes, and improve socioeconomic outcomes [3,9,10].

While prior studies, including some systematic reviews, have found that social protection interventions can improve TB treatment outcomes, [11] they were limited in their ability to widely inform policy and programmatic implementation of various types of social protections in a wide array of settings. De Andrade et al., for example, limited their meta-analysis to randomized control trials [12]. While high quality, these results exclude a substantial body of evidence generated by high quality observational studies on a range of TB treatment outcomes. Another study conducted by Richterman et al. focused only on cash transfers, thereby not including studies that describe other types of social protection interventions such as food assistance or psychosocial support.^11^ No systematic review has described the impact of social protection interventions on socioeconomic outcomes, including catastrophic costs, dissaving, or standardized measures of poverty. The objective of this systematic review and meta-analysis is to provide synthesized evidence of health and socioeconomic effectiveness of social protection interventions for TB-affected individuals and households required to inform global scale-up of these strategies.

## Methods

Our systematic review and meta-analysis were conducted in accordance with PRISMA guidelines. Specifically, we 1) iteratively developed a list of search terms and a search strategy, 2) searched for relevant articles in a pre-determined number of databases, 3) systematically screened titles and abstracts for potentially eligible articles, 4) read the full text of articles deemed eligible for full text review, 5) agreed upon articles that met criteria for inclusion, 6) extracted relevant data from each study, 7) conducted quality and risk of bias assessments, and 8) synthesized available evidence.

We used the Population, Intervention, Comparison, Outcome, and Time (PICOT) format to define our research questions for this systematic review and meta-analysis (S1 Text):

1. Do people with TB who have enrolled in at least one social protection intervention demonstrate an improvement in TB treatment success (treatment completion or cure) compared to people with TB who have not enrolled in and/or been recipients of social protection interventions?
2. Do people with TB who have enrolled in at least one social protection intervention have better socioeconomic outcomes, including lower rates of catastrophic costs and dissaving, compared to people with TB who have not enrolled in and/or been recipients of social protection interventions?

This systematic review protocol was guided by the Preferred Reporting Items for Systematic Reviews and Meta-Analyses (PRISMA) protocol checklist [13]. A scoping review [14] was also conducted to landscape the available evidence and to inform the protocol for this analysis, which has been published previously [15].

### Search strategy and selection criteria

Our initial search was conducted March 2021, and then repeated September 2023, January 2025, and December 2025 to include articles published through December 2025. We included three electronic databases: PubMed (includes MEDLINE), Embase, and Web of Science. We used Google Scholar Advanced to search selected, relevant databases, explained in detail in our scoping review [14]. Articles were imported into Covidence [16] for systematic screening by the study team (S2 Text) in accordance with PRISMA guidelines. Articles deemed eligible for inclusion were reviewed by four researchers (MH, HT, AS and CC; S2 Text).

We included randomized controlled trials, cross-sectional studies and cohort studies. Studies were included if they described people with pulmonary and extra pulmonary TB, people with drug-sensitive (DS-TB) and drug-resistant (DR-TB)/multi-drug resistant TB (MDR-TB), people either with or without HIV-TB co-infection, or TB-affected households in either low-to-middle-income countries (LMICs) and/or high burden TB countries as defined by WHO or the World Bank [1,17]. We only included studies published since 2012, which coincided with the “World Bank’s Social Protection and Labour Strategy 2012-2022,” [18] which supported initiatives on reducing socioeconomic risk, after which definitions of social protection were expected to be uniform. We did not restrict our search by language.

### Intervention

We only included studies in which the main independent variable was enrollment in, and/or receipt of, a social protection intervention as defined by the World Bank [5]. We included social protection interventions specifically designed for individuals or households affected by TB (*TB-specific social protection interventions*), for which microbiologic or clinical diagnosis of TB is a requirement of eligibility for the social protection intervention, as well as social protection interventions designed for individuals or households inclusive of those affected by TB but identified as eligible based on non-TB criteria (*TB-sensitive social protection interventions*) [19].

We excluded interventions that were not intended to reduce socioeconomic risk; for example, nutritional support as a clinical intervention during a hospitalization was not considered to be social protection, and studies describing these types of intervention were not included.

### Outcomes

We only included studies for which the main dependent variable was at least one standardized outcome related to TB treatment including cure, treatment completion, treatment success (a composite variable of TB cure and treatment completion) mortality/death, treatment default, loss to follow up, [1] and/or at least one standardized socioeconomic outcome such as catastrophic costs, dissaving or impoverishment, in accordance with global definitions [1].

### Screening and descriptive analysis

**Fig 1:**
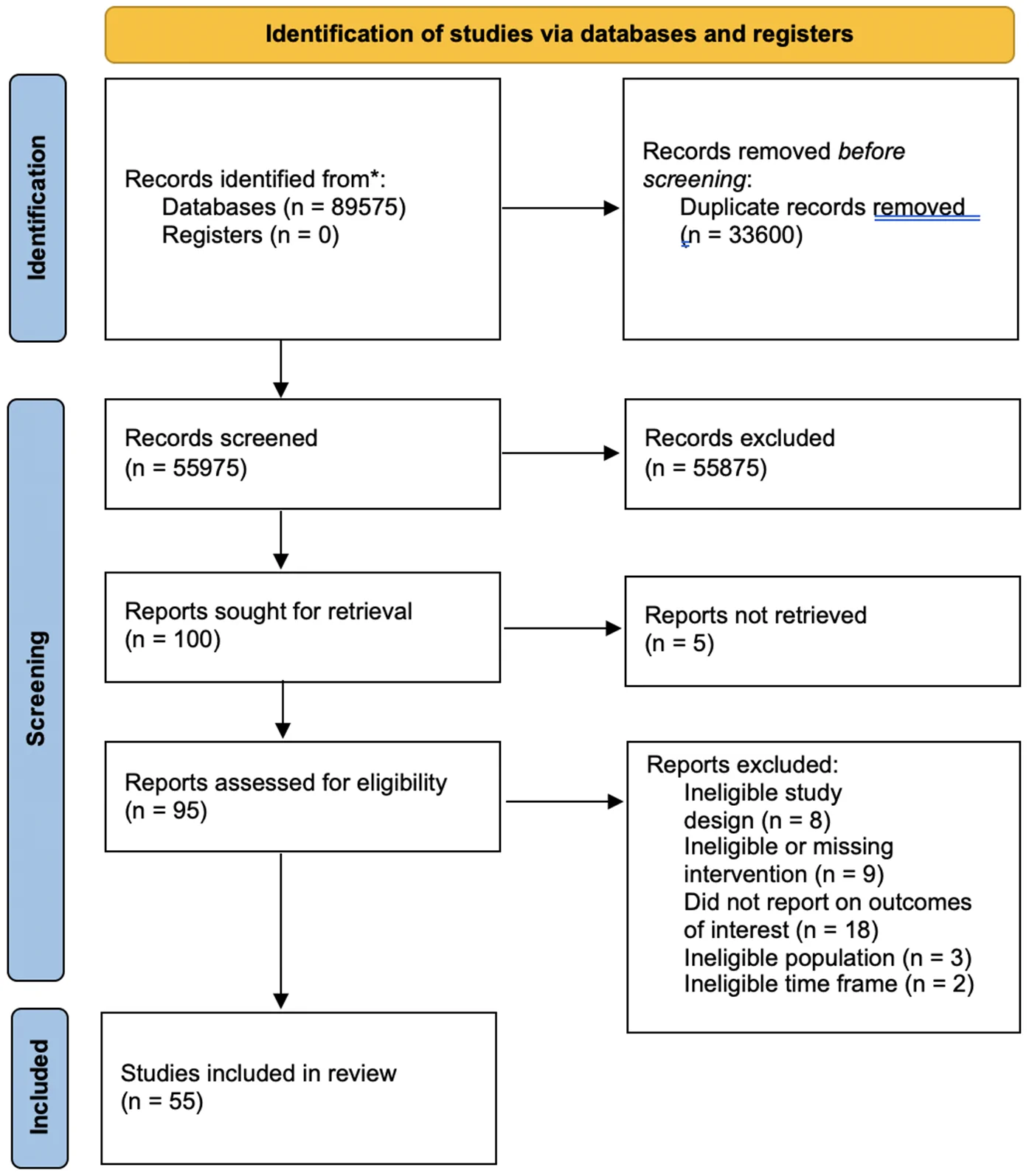
Flow diagram of search process to identify eligible studies using PRISMA.

Titles and abstracts were systematically and independently screened by the study team (S2 Text). Potentially eligible articles underwent full text review. Questions about study inclusion were discussed by reviewers (MH, HT) with adjudication by a third reviewer (TN) or senior researchers (TW, PBS). Fig 1 describes the search process in accordance with PRISMA protocols for systematic reviews.

### Quantitative analysis

Three reviewers (MH, AS, CC) independently extracted quantitative data reported in eligible papers (S1-S4 Tables). For the meta-analysis, authors extracted data describing the number or percent of individuals who attained TB treatment success in both the treatment (receipt of social protection in conjunction with standard biomedical treatment) and the control (standard biomedical treatment only) groups. All estimates of effect for dichotomous outcomes (e.g., “achieved treatment success” versus “did not achieve treatment success”) were reported as odds ratios with a 95% confidence interval. We used a random effects model [20] for our meta-analysis to account for heterogeneity between studies. For our subgroup analyses, authors extracted data describing type of social protection intervention, study setting, WHO region, and whether an intervention was considered TB sensitive or TB specific. All calculations were conducted in STATA SE version 19 [21].

A logistic meta-regression was performed to evaluate the association of study characteristics with the odds of TB treatment success. We selected a limited number of study characteristics to not overfit the model including: 1) study quality, 2) whether the study included individuals with MDR-TB, and 3) study setting. Study quality for studies was scored using the Newcastle Ottawa Scale (NOS); a summed score of 9–8 was categorized as a high-quality study; a summed score of 7–4 (inclusive) was categorized as a medium quality study, and a summed score of 3–0 (inclusive) was categorized as a low-quality study. Risk of bias assessments are summarized in S5 and S6 Tables.

### Qualitative analysis

We reviewed qualitative manuscripts according to theme and content, and only included papers that described either TB treatment or socioeconomic outcomes. Findings are summarized in S2 Table.

### Risk of bias in individual studies

We used the Cochrane Risk of Bias (RoB) tool [22] for RCTs and the Newcastle Ottawa Scale [23] (NOS) for all studies that quantitatively reported on outcomes. Studies are scored in 3 areas – selection (scored 0–4), comparability (scored 0–2), and outcome (scored 0–3), with higher scores indicating a positive assessment (less likely to be biased). Risk of bias was appraised by five researchers independently (MH, HT, AS, CC, PBS) (S5 and S6 Tables).

### Role of the funder

Funding for this study comes from the Nina Ireland Program in Lung Health (PI: Shete, fund number 7710-138404-7504523-45). The funder of the study had no role in study design, data collection, data analysis, data interpretation, or writing of the report.

## Results

After removing duplicates, we screened 55,975 titles and abstracts (Fig 1). After removing 55,880 articles based on title and abstracts, we evaluated 95 articles for eligibility. Forty-one articles did not meet our criteria for eligibility (S8 Table). Of the 55 included articles, 51 articles reported primarily on quantitative outcomes, while four articles reported only on qualitative outcomes [24–27]. The results of our search are reported in Fig 1 in accordance with PRISMA guidelines. Study characteristics including interventions, outcomes, and setting are summarized in S1-S4 Tables.

Of the 51 articles that reported on quantitative outcomes, 42 (82.4%) were cohort studies, three studies (5.9%) were cross-sectional, one study (2%) was quasi-experimental, one (2%) was a randomized controlled trial, and four studies (7.8%) were cluster randomized trials. No studies were ecological. Studies were conducted across 18 different countries (S1 Table and S2 Table) and across urban, rural, and mixed settings (S1 & S2 Tables). Six different categories of social protection interventions were described in the studies that reported quantitative outcomes (S3 Table), with cash transfers (n = 25, 49%) being the most frequently described. Social protection interventions described in the qualitative studies reviewed (n = 4) included psychosocial, nutritional, and financial support in the form of transportation stipends. Interventions were described as either TB sensitive, e.g., eligibility criteria primarily pertaining to measures of poverty, or TB specific, for which only TB affected individuals and/or households were eligible. Thirty studies reported TB treatment success as the primary outcome of interest. Other outcomes included were mortality, TB treatment default (treatment interruption of at least two months [28], loss to follow up, treatment failure, or a combination of TB treatment outcomes (S4 Table) [29].

Eight studies reported primarily on socioeconomic outcomes, while two studies reported secondary socioeconomic outcomes (S4 Table). Five papers described how social protection reduced the risk of incurring catastrophic costs [3,30–33]. Wingfield et al., for example, found that a conditional cash transfer defrayed 20% of total costs, 39% of direct costs, and 19% of lost income. Among poorer households and female participants, the cash transfer defrayed total costs 22% and 23%, respectively [3]. Similarly, Florentino et al. found that social protection reduced catastrophic costs to a much greater extent among individuals with drug-resistant TB, who incurred higher costs than individuals with drug-susceptible TB [32]. As a secondary outcome, Li et al. found that expenses related to hospital admissions decreased as a result of social protection [34]. Three studies found that social protection did not improve outcomes, [35–37] while Liu et al. found that outpatient TB treatment outcomes improved with social protection while inpatient TB treatment outcomes were unchanged [38].

The majority of studies did not describe implementation process metrics, namely fidelity, reach, and coverage (S4 Table). Reasons for poor implementation fidelity reported by nine studies were related to administrative issues such as inadequate banking systems and/or delayed or non-disbursement of social protection benefits. Fidelity, coverage, or feasibility of social protection interventions was not consistently reported, which precluded analysis of implementation outcomes.

Results of the risk of bias assessments are described in S5 Table and S6 Table. AS, CC, MH and PS used the Cochrane RoB Tool for Randomized studies, [22] and the NOS [39] to independently evaluate risk of bias in non-randomized studies that reported quantitatively on outcomes. We found the quality of the studies included to be high based on criteria specified in the Cochrane RoB tool and the NOS. Assessments of certainty and the quality of evidence of across studies were evaluated using the Grading of Recommendations, Assessment, Development, and Evaluation (GRADE) (further described in S9 Table).

Random-effects meta-analysis was used for our primary outcome of interest, TB treatment success. Of the 33 studies that quantitatively reported on TB treatment success, two studies did not have a control group [38,40] and three studies did not report findings such that data could be extracted for meta-analysis calculations [41–43]. One study did not provide information about how the historical cohort was recruited and compared to the intervention cohort [44]. Lastly, although Wrohan et al. [45] reported treatment success by social health insurance status, researchers only evaluated one type of social health insurance as the “social protection intervention,” and did not include additional information about the cohort of individuals not using social health insurance. Authors of this meta-analysis agreed that there was not sufficient information provided in the study to distinguish the intervention from the control cohort. In addition, to optimize clarity, we have included S7 Table which summarizes the rationales for exclusion from the meta-analysis. We therefore included 26 studies in our meta-analysis.

Lastly, one study included in the meta-analysis described how although a larger cohort of individuals was enrolled in a social protection intervention, only 233 individuals actually received the disbursed funds [46]. Authors therefore modified their analytic approach according to how many individuals actually received disbursed funds; [46] thus, we included only those who were enrolled and received the intervention in our meta-analysis. Studies that had a significant number of participants lost to follow up were scored accordingly in our risk of bias assessments (S5 Table and S6 Table).

**Fig 2:**
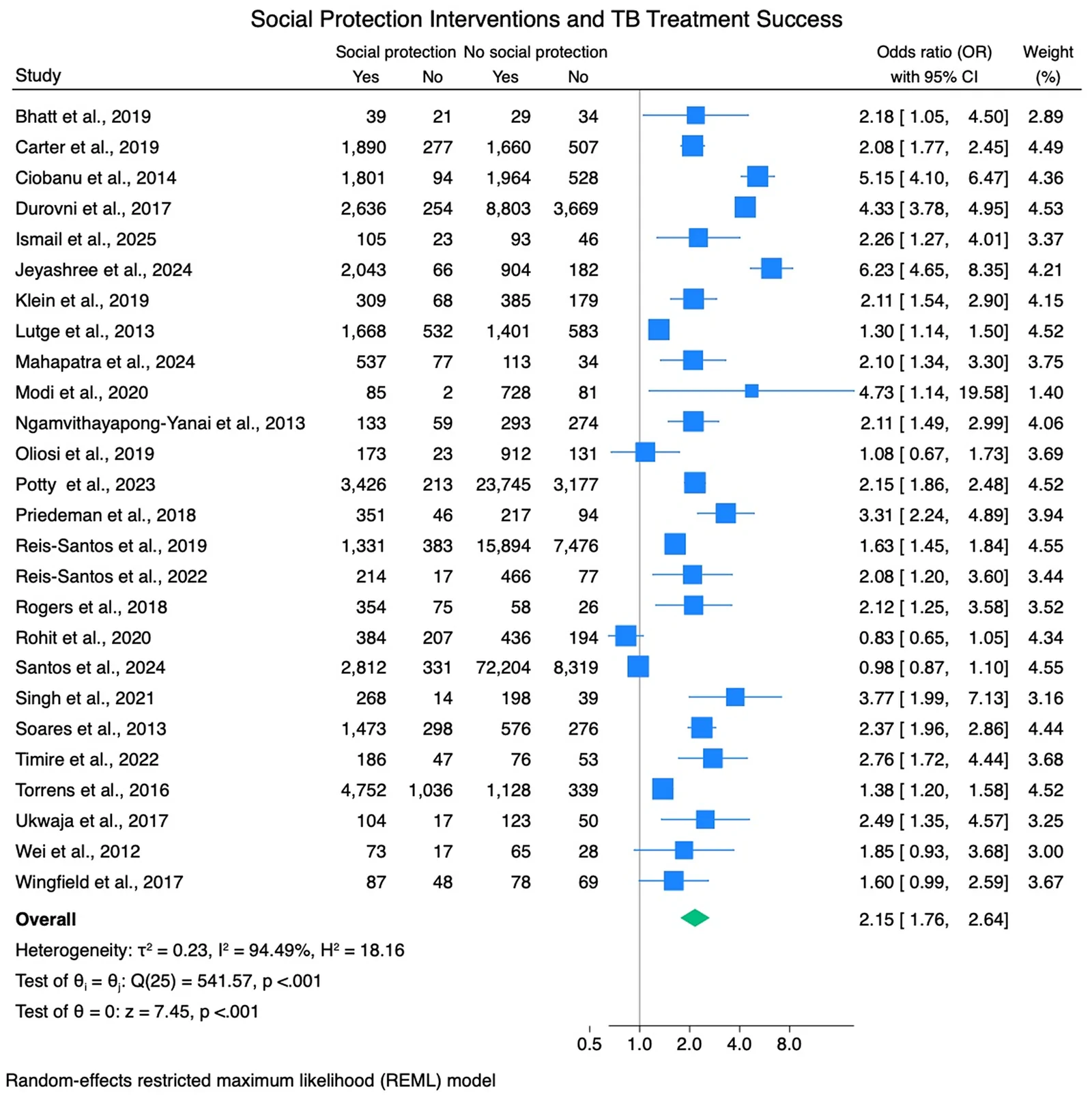
Meta-analysis of treatment success rates in individuals who were enrolled and/or recipients of social protection vs. those who were not enrolled and/or recipients of a social protection intervention. Treatment = exposed to social protection, control = not exposed to social protection. The three RCTs included in the meta-analysis were Ismail et al. (2025), Reis-Santos et al., (2019) and Wingfield et al. (2017).

People with TB who were exposed to social protection interventions in conjunction with standard biomedical treatment had approximately two times the odds of achieving TB treatment success (Fig 2). The distribution of study effects is relatively narrow, with almost all studies reporting a positive effect. Our pooled estimate has an odds ratio of 2.15 (95% CI 1.76 to 2.64, p < .001), indicating that social protection was significantly associated with TB treatment success. However, the I^2^ of 94.49% suggests high between-study heterogeneity.

To better characterize the range of true effects, we also reported the 95% prediction interval (OR 0.71- 6.53) (S1 Fig). The prediction interval indicates that on average across multiple studies, social protection increased the odds of TB treatment success, but the extent of the effect of social protection could range from null to substantial.

The odds of TB treatment success were regressed on study covariates as shown in Table 1. Covariates were chosen based on previously published studies indicating that study quality, [47] inclusion of individuals with MDR-TB, [48] study setting, [9] and type of social protection [9] can influence the effect of an intervention. The results of our meta-regression indicated that conducting a study in a rural setting, the inclusion of MDR-TB, and a non-cash social protection intervention type were all not significantly associated with lower study odds of TB treatment success. Adjusting for other variables, the relationship between study quality and study odds ratio was not statistically significant; however, there was a trend for lower odds ratios of treatment success in high quality (-1.8 [-5.1, 1.6]) and medium quality (-2.3 [-7.4, 2.7]) studies compared to low quality studies.

**Table 1:** Regression coefficients and p values from meta-regression of the study odds of TB treatment success across covariates of interest (n = 26).

Variable	Regression coefficient (95% CI)	P value
Study quality		
*Low quality (reference)*	--	--
*Medium quality*	-2.3 (-7.4, 2.7)	0.3
*High quality*	-1.8 (-5.1, 1.6)	0.3
Inclusion of individuals with MDR TB		
*Yes*	1 (-1.5, 3.6)	0.4
*No*	0.8 (-1.8, 3.3)	0.5
Study setting		
*Primarily rural*	-.2 (-5, 4.5)	0.9
*Mixed urban/rural*	-.4 (-3.3, 2.6)	0.8
*Primarily urban*	-.5 (-3.6, 2.5)	0.7
Social protection type		
*Cash (reference)*	--	--
*Nutrition*	.6 (-2.1, 3.4)	0.6
*Psychosocial*	-.1 (-2.8, 2.5)	0.9
*Mix of interventions*	.3 (-16, 2.3)	0.7

To evaluate the robustness of our findings and to elucidate the sources of heterogeneity, we conducted two additional types of analyses: 1) a leave-one-out sensitivity analysis, 2) subgroup analyses by intervention type, study setting, geographic region, and whether or not an intervention was TB specific or sensitive.

Leave-one-out analysis (S2 Fig) suggests that high heterogeneity could not be attributed to a single study; the pooled estimate remained stable (2.05-2.25) and statistically significant across all analyses (p < .001 across all iterations). This finding suggests that the heterogeneity cannot be attributed to a single outlier study.

Subgroup analysis by intervention type demonstrated that cash transfers, nutritional support, psychosocial support, and mixed interventions all increased the odds of TB treatment success (OR 1.95, 2.41, 2.23, and 2.41, respectively, p < .001 for all subgroups) (S3 Fig). However, the test for subgroup differences was not statistically significant (Qb(3)=1.75, p = 0.63), suggesting that the high heterogeneity across all studies could not be attributed to various types of social protection interventions. Similarly, study setting did not appear to be a source of heterogeneity (Qb(3)=0.84, p = 0.84), as social protection was associated with TB treatment success across primarily urban, primarily rural, and mixed settings (S4 Fig). There was significant variability in TB treatment success across different WHO regions (Qb(4)=11.57, p = 0.02), with pooled ORs ranging from 1.81 in the Americas to 4.25 in the European Region (S5 Fig). TB specific interventions had significantly higher odds of TB treatment success (OR 2.40, 95% CI 1.91 to 3.02) compared to TB sensitive programs (OR 1.43, 95% CI 1.04 to 1.95), and there was a statistically significant difference between the two subgroups (Qb(2)=6.98, p = 0.03) (S6 Fig).

Four eligible studies utilized qualitative methods to describe the impact of social protection interventions. While the number of studies were too few to provide a meaningful content analysis, we identified themes across studies. Primarily, we evaluated qualitative studies according to type of social protection intervention and actual or perceived success or non-benefit of an intervention. We found that social protection interventions generally had high levels of acceptability among health care workers and people affected by TB, [24,25,49] and that interventions that offset the costs of direct non-medical costs related to TB enabled treatment adherence [49,50]. However, no studies explicitly evaluated potential negative perceptions or barriers to social protection.

## Discussion

The results of this systematic review and meta-analysis provide a holistic assessment of the evidence available on the effectiveness of social protection interventions on TB treatment outcomes. Our search yielded 55 studies overall, including 26 studies eligible for a meta-analysis demonstrating that social protection interventions, when administered to individuals affected by TB and in conjunction with standard of care treatment, can double the odds of TB treatment success. This finding suggests that social protection interventions are associated with substantially improved TB treatment outcomes for TB affected individuals. While large seminal papers such as Jesus et al. (2025) [51] and Bhargava et al. (2023) [40] found that social protection can significantly reduce TB incidence and mortality, our systematic review highlights a larger body of evidence demonstrating that social protection can significantly improve TB treatment and socioeconomic outcomes. These results support the need to scale-up these programs globally in keeping with recent UN General Assembly High Level meeting commitments to the fight against TB which calls for all TB-affected individuals to have access to social protection interventions to prevent financial hardship by 2027 [7].

The findings of our meta-analysis, and our subsequently sub-group analysis, provide insight into the high variability across studies. While our pooled OR of 2.15 was relatively robust (95% CI 1.76 to 2.64, p < .001), this result represents an average effect across a wide range of studies that include different geographic settings, social protection interventions, and populations. Our leave-one-out analysis confirmed that no single study drove the variability, and that the pooled estimate was stable across each iteration of the analysis. However, the high heterogeneity and wide prediction interval, which better estimates the range of effects across different study settings and populations compared to the pooled OR, indicated that the magnitude of the benefit can range considerably across different contexts. This finding warranted further analysis to elucidate sources that drove heterogeneity.

Our subgroup analyses revealed two sources of between-study heterogeneity. First, WHO region proved to be a significant source of between-study heterogeneity. These findings suggest that the magnitude of the effect of social protection can vary substantially given the sociodemographic features of a population, baseline TB epidemiology, and effective implementation of social protection interventions. Second, whether an intervention was TB specific or TB sensitive was also a significant source of between-study heterogeneity, with TB-specific programs conferring a much greater effect on TB treatment success. These findings parallel those by Ferreira et al. [52], and are perhaps the most significant in terms of health and social welfare policy. Albeit moderate, TB-sensitive social protection interventions still significantly improve the odds of TB treatment success, despite not being targeted specifically towards individuals with TB. Countries with existing, robust TB-sensitive interventions may consider interventions to ensure that TB-affected individuals have access to existing social protection interventions. TB specific interventions, which may be less costly, can confer a much greater benefit. National TB programs should consider incorporating social protection interventions to ensure higher rates of TB treatment success.

Our findings demonstrated similar results to prior systematic reviews. For example, the 2018 study conducted by Richterman et al. [11] found that cash transfer interventions for TB-affected individuals was associated with 1.77 times the odds of a “positive TB treatment outcome.”^11^ The 2018 systematic review and meta-analysis by Andrade et al. found that participants who were beneficiaries of social protection interventions had 1.09 times the odds of TB treatment success and 1.11 times the odds of a cure compared to those who were not beneficiaries of social protection interventions [12]. While these prior studies are informative, we aimed to be more expansive and consistent in the definitions used for this review to align with global guidance on implementation of social protection for TB-affected households [53]. In particular, our analysis was based on current comprehensive definitions of social protection as endorsed by the World Bank and International Labor Organization (S3 Text) [5,54]. Although this search strategy yielded many articles that did not meet eligibility criteria, we captured studies that we may not have otherwise found with a narrower definition of social protection. Our subgroup analyses helped categorize these interventions by both type (cash transfer, food assistance) and whether the intervention was TB specific or sensitive.

Our study also sought to estimate the potential impact of social protection interventions on reducing the risk of catastrophic and out-of-pocket costs among TB-affected households [3,24,30]. However, the types of socioeconomic outcomes reported on by eligible studies were variable and not comprehensive. For example, only five studies reported on the socioeconomic outcomes we specified in our search criteria; no studies reported on impoverishment measures. Although we aimed to provide a comprehensive assessment of the impact of social protection on socioeconomic outcomes, our outcomes of interest were underrepresented and insufficient to synthesize. The limitations of these findings highlight the need for standardizing socioeconomic outcomes for future studies in a manner that supports programmatic monitoring and evaluation of global indicators. Further, such findings would be key to developing person-and household centered approaches to developing and implementing social protection interventions to improve socioeconomic outcomes.

Operational challenges to providing social protection feasibly and sustainably for TB-affected individuals have long been cited as a barrier for program coverage and uptake [55]. We analyzed eligible studies to inform operational and implementation outcomes including feasibility, coverage, or uptake. Unfortunately, the majority of studies did not describe these implementation outcomes or did not use standardized metrics (S4 Table). Of studies which reported implementation challenges, several describe administrative hurdles to provision of benefits; for example, unreliable banking systems resulting in delayed or failed disbursement of funds to beneficiaries and mitigating the potential impact of the intervention (S4 Table). Additional research to identify barriers and facilitators to accessing social protection, as well as standardizing and reporting implementation outcomes, would provide needed evidence on feasibility, acceptability, and coverage necessary to support the deployment, monitoring, and scale up of interventions.

While the methods utilized in this systematic review, including robust risk of bias assessment and meta-regression, enhance our findings, our approach has limitations. The majority of studies that met eligibility criteria were from middle income settings, primarily Brazil, limiting generalizability of our findings; half of the high burden TB countries are located in Africa, are predominantly classified as low-income, and lack the robust landscape of existing social protection programs that Brazil has [28]. Second, the majority of studies reported cash transfer interventions. Additional evidence about other types of social protection interventions would be beneficial, especially as National TB Programs and other key stakeholders begin designing, implementing, and scaling up interventions. Lastly, because the majority of studies included in the meta-analysis were observational, we cannot exclude residual confounding.

In conclusion, social protection interventions have the potential to significantly improve TB treatment outcomes. Our meta-analysis and subgroup analysis indicate that, on average, individuals who had access to social protection interventions had twice the odds of attaining TB treatment success compared to those who did not have access to social protection interventions. Our results, which are grounded in high quality evidence and robust to bias based on our meta-regression and subgroup analyses, indicate that a wide variety of social protection interventions can be considered to improve both TB treatment and socioeconomic outcomes. The standardized outcomes and definitions used in this systematic review and meta-analysis have the potential to guide further research on social protection programs for TB-affected populations.

### Patient and public involvement

It was not appropriate or possible to involve patients or the public in the design, or conduct, or reporting, or dissemination plans of our research.

## Supporting information

S1 Text PICOT framework. (DOCX)

S2 Text Study team. (DOCX)

S3 Text Outcomes by PICOT. (DOCX)

S4 Text Search strategy keywords. (DOCX)

S5 Text Search strategies. (DOCX)

S1 Fig Forest plot of the random-effects meta-analysis with 95% prediction interval. (TIF)

S2 Fig Leave-one-out sensitivity analysis for the random-effects meta-analysis. (TIF)

S3 Fig Subgroup analysis by intervention type. (TIF)

S4 Fig Subgroup analysis by study setting. (TIF)

S5 Fig Subgroup analysis by WHO region. (TIF)

S6 Fig Subgroup analysis of TB sensitive and TB specific social protection interventions. (TIF)

S1 Table Study characteristics of quantitative studies (n = 51). (DOCX)

S2 Table Study characteristics of qualitative studies (n = 4). (DOCX)

S3 Table Social protection interventions described in quantitative studies (n = 51). (DOCX)

S4 Table Outcomes and implementation challenges described in quantitative studies (n = 46). (DOCX)

S5 Table Risk of bias assessments of non-randomized studies using the Newcastle Ottawa Scale (NOS). (DOCX)

S6 Table Risk of bias assessments of randomized controlled trials (RCTs) using the Cochrane Risk of Bias (RoB) assessment tool. (DOCX)

S7 Table Studies that reported on TB treatment success as the primary outcome of interest, but were not included in the meta-analysis. (DOCX)

S8 Table A listing of excluded studies (n = 40). (DOCX)

S9 Table GRADE assessment of certainty of evidence. (DOCX)

S10 Table Listing of all studies, both included and excluded, with reasons for exclusion if applicable. (DOCX)

S1 Data All extracted data from included studies. (XLSX)

S2 Data All extracted data required to conduct meta-analysis, meta-regression, and subgroup analyses. (XLSX)

S1 File PRISMA Checklist. Page MJ, McKenzie JE, Bossuyt PM, Boutron I, Hoffmann TC, Mulrow CD, et al. The PRISMA 2020 statement: an updated guideline for reporting systematic reviews. BMJ 2021;372:n71. https://doi.org/10.1136/bmj.n71. Licensed under CC BY 4.0. To view a copy of this license, visit https://creativecommons.org/licenses/by/4.0/
(DOCX)
