# Supplementary material for: The impact of social protection interventions on treatment and socioeconomic outcomes of tuberculosis-affected people and households in low income, high burden settings: A systematic review and meta-analysis
Source: PLOS Glob Public Health. 2026 Sep 3;6(9):e0006704. doi: 10.1371/journal.pgph.0006704 (PMC13541142; doi:10.1371/journal.pgph.0006704)
Supplement: Supplementary file 1 [file pgph.0006704.s027.pdf]

Center for
Tuberculosis
UCSF
University of California
San Francisco

ctb@ucsf.edu
www.tb.ucsf.edu

July 28, 2026

Dear Editor and Reviewers,

First and foremost, we are very grateful to the reviewers and thank them for their additional insightful and constructive comments, which have further strengthened the manuscript to make it suitable for publication in ***PLOS Global Public Health***. Below, we have addressed each point made by both reviewers.

1. We thank both Reviewers 1 and 2 for pointing out the descrepancy between the abstract and the manuscript describing the total number of studies included in the meta-analysis. We have modified our manuscript (page 12, line 305):

"*We therefore included 26 studies in our meta-analysis.*"

2. We are grateful to Reviewer 1 for their incredible attention to detail in noting grammatical errors throughout the text. We have made the following changes to the manuscript:

"…reduced catastrophic costs to a much greater extent among individuals with drug-resistant TB" (page 11, line 273)

"Our search yielded 55 studies overall, including 26 studies eligible for a meta-analysis demonstrating that social protection interventions, when administered to individuals affected by TB and in conjunction with standard of care treatment, can double the odds of TB treatment success" (page 15, lines 392-395)

"…and there was a statistically significant difference between the two subgroups." (page 14, line 373)

"Specifically, we 1) iteratively developed a list of search terms and a search strategy…" (page 5, line 128)

"We found that social protection interventions generally had high levels of acceptability among health care workers and people affected by TB" (page 15, lines 383-384)

3. We agree with Reviewer 1 that our previously stated assessment of the impact of social protection on TB treatment outcomes was overstated. Per the recommendation of Reviewer 1, we have modified our manuscript accordingly:

"This finding suggests that social protection interventions are associated with substantially improved TB treatment outcomes for TB affected individuals." (page 15, lines 395-396)

We have also reviewed the manuscript in its entirety to address any additional grammatical errors and typos.

4. We thank Reviewer 1 for their thoughtful comment about acknowledging publication bias and clarifying the importance of a prediction interval relative to a pooled OR. We have therefore modified our discussion accordingly.

Center for Tuberculosis
UCSF
University of California San Francisco

ctb@ucsf.edu
www.tb.ucsf.edu

"However, the high heterogeneity and wide prediction interval, which better estimates the range of effects across different study settings and populations compared to the pooled OR, indicated that the magnitude of the benefit can range considerably across different contexts. This finding warranted further analysis to elucidate sources that drove heterogeneity." (page 16, lines 420-424)

"Lastly, because the majority of studies included in the meta-analysis were observational, we cannot exclude residual confounding." (page 18, lines 491-492)

5. We appreciate Reviewer 2's attention to detail and their comment that we needed to clarify the text pertaining to the exclusion of studies describing specific types of interventions. We have modified our text accordingly to clarify that we did not include clinical support interventions:

"We excluded interventions that were not intended to reduce socioeconomic risk; for example, nutritional support as a clinical intervention during a hospitalization was not considered to be social protection, and studies describing these types of intervention were not included." (page 7, lines 179-181)

6. We again thank Reviewer 2 for their careful attention to detail, and for noticing that we had listed "intervention type" twice in our description of subgroup analyses. The manuscript now states:

"To evaluate the robustness of our findings and to elucidate the sources of heterogeneity, we conducted two additional types of analyses: 1) a leave-one-out sensitivity analysis, 2) subgroup analyses by intervention type, study setting, geographic region, and whether or not an intervention was TB specific or sensitive." (page 14, lines 353-356)

7. We appreciate that Reviewer 2 noticed we had incorrectly referenced a first author. We have modified our manuscript accordingly:

"These findings parallel those by Ferriera et al.[54], and are perhaps the most significant in terms of health and social welfare policy" (page 16, lines 431-433).

We again thank the reviewers and editors for their thoughtful, detailed, insightful responses. We greatly appreciate the opportunity to revise our manuscript.

Sincerely, on behalf of all authors,

Mollie Hudson, PhD, MSN, MS, NP-C
Assistant Adjunct Professor
Department of Social and Behavioral Sciences, School of Nursing Scholar, Tuberculosis Research and Mentoring Program
Center for Tuberculosis
University of California, San Francisco
