# Supplementary material for: The impact of social protection interventions on treatment and socioeconomic outcomes of tuberculosis-affected people and households in low income, high burden settings: A systematic review and meta-analysis
Source: PLOS Glob Public Health. 2026 Sep 3;6(9):e0006704. doi: 10.1371/journal.pgph.0006704 (PMC13541142; doi:10.1371/journal.pgph.0006704)
Supplement: Supplementary file 2 [file pgph.0006704.s001.docx]

**S1 Text.** PICOT framework

| **PICOT statement** | **Population** | **Intervention** | **Comparison** | **Outcome** | **Time** |
| --- | --- | --- | --- | --- | --- |
| #1 | TB-affected individuals and households | Social protection in conjunction with standard biomedical TB treatment (medication to treat TB, standard medical appointments) | Individuals who are not recipients of/enrolled in social protection; biomedical TB treatment only | TB treatment outcomes (cure, treatment completion, death, treatment default, TB treatment success) | 2012-2023 |
| #2 | TB-affected individuals and households | Social protection in conjunction with standard biomedical TB treatment (medication to treat TB, standard medical appointments) | Individuals who are not recipients of/enrolled in social protection; biomedical TB treatment only | Improved socioeconomic outcomes (e.g. reduced catastrophic costs, dissaving, various measures of poverty) | 2012-2023 |
