# Supplementary material for: The impact of social protection interventions on treatment and socioeconomic outcomes of tuberculosis-affected people and households in low income, high burden settings: A systematic review and meta-analysis
Source: PLOS Glob Public Health. 2026 Sep 3;6(9):e0006704. doi: 10.1371/journal.pgph.0006704 (PMC13541142; doi:10.1371/journal.pgph.0006704)
Supplement: Supplementary file 3 [file pgph.0006704.s002.docx]

**S2 Text.** Study team

| **Name** | **Initials** | **Role** |
| --- | --- | --- |
| Mollie Hudson | MH | Conceptualization, title and abstract screening, full text review, data extraction, meta-analysis, writing |
| Heather Todd | HT | Conceptualization, title and abstract screening, full text review, data extraction, meta-analysis, writing |
| Delia Boccia | DB | Conceptualization |
| Canice Christian | CC | Title and abstract screening, full text review, data extraction, risk of bias assessments |
| Joseph Kazibwe | JK | Title & abstract screening |
| Talemwa Nalugwa | TN | Conceptualization |
| Joseph Pearman | JP | Title & abstract screening |
| Shreya Puntambekar | SP | Full text review, data extraction |
| Ann Schraufnagel | AS | Data extraction, risk of bias assessments, meta-analysis |
| Priya B. Shete | PBS | Conceptualization, full text review, data extraction, meta-analysis, writing |
| Kristina Skender | KS | Title & abstract screening |
| Phuong Tran | PT | Title & abstract screening |
| Tom Wingfield | TW | Conceptualization, full text review, data extraction, meta-analysis, writing |
