# Supplementary material for: The impact of social protection interventions on treatment and socioeconomic outcomes of tuberculosis-affected people and households in low income, high burden settings: A systematic review and meta-analysis
Source: PLOS Glob Public Health. 2026 Sep 3;6(9):e0006704. doi: 10.1371/journal.pgph.0006704 (PMC13541142; doi:10.1371/journal.pgph.0006704)
Supplement: Supplementary file 4 [file pgph.0006704.s003.docx]

**S3 Text.** Outcomes by PICOT

- **Outcomes for PICOT #1:** Primary and secondary outcomes related to TB treatment and catastrophic costs, and the nature of the social protection intervention.
  - *Primary TB treatment outcome:*
    - TB treatment success
    - Death
  - *Secondary TB treatment outcomes:*
    - Cure
    - Treatment completion
    - Adverse TB treatment outcomes:
      - Loss to follow up
      - Relapse
      - Treatment failure
        - While this terminology as no longer used, it is likely that studies will have used this terminology.
      - No evaluation
- **Outcomes for PICOT #2:**
  - Catastrophic costs
    - Catastrophic costs (total costs of entire TB illness >20% of the same household’s annual pre-TB income)
    - Costs
      - Direct medical
      - Direct non-medical
      - Indirect (lost income, time, and productivity)
        - Of note, these metrics may be calculated different based on the study approach, which will have to be taken into account when analyzing our findings.
  - Dissaving
    - Dissaving
      - If the patient/household took out a formal or informal loan
      - If the patient/household sold an asset or item
      - If the patient/household used savings
      - If the patient/household took a child out of school
      - Reduced household food consumption
  - Percent poor based on multidimensional poverty index scores (reference: World Bank Group. *Reversals of Fortune: Poverty and Shared Prosperity 2020*.; 2020. doi:10.1038/302765a0)
    - Percent poorer than median poverty score (person with TB and/or TB-affected household)
      - Experiencing extreme poverty
      - Below specified higher poverty lines (USD $3.20 or $5.50 (TB-affected household)
      - % below SPL (TB-affected household)
    - Person with TB and/or TB-affected household’s perception of poverty and the impact of TB on their poverty
      - For example, if a study used the WHO TB Patient Cost Survey, which asks questions about how TB illness has affected individual and/or household level poverty
