# Supplementary material for: The impact of social protection interventions on treatment and socioeconomic outcomes of tuberculosis-affected people and households in low income, high burden settings: A systematic review and meta-analysis
Source: PLOS Glob Public Health. 2026 Sep 3;6(9):e0006704. doi: 10.1371/journal.pgph.0006704 (PMC13541142; doi:10.1371/journal.pgph.0006704)
Supplement: Supplementary file 5 [file pgph.0006704.s004.docx]

**S4 Text.** Search strategy keywords

|  | **Generic keywords** | **Other keywords** |
| --- | --- | --- |
| **1** | Tuberculosis (“TB treatment terms) | TB, Mycobacterium tuberculosis, pulmonary TB,  TB-affected, TB-infected, TB patients, drug-resistant TB, TB individuals/households, TB prevalent, TB cases Pulmonary TB, PTB |
| **2** | social protection (“intervention terms”) | Social safety net  Socioeconomic support  Social support  Economic support  Financial support  Cash transfers; food-based programs, supplementary feeding programmes, food stamps, vouchers, and coupons; in-kind transfers such as school supplies and uniforms; conditional cash transfers; price subsidies for food, electricity, or public transport; public works programmes; and fee waivers and exemptions for health care, schooling, and utilities, welfare  Food baskets, food rations  Protections against shocks  Social risk management  Transportation  Government financing  Reimbursement  Low and middle income, LMIC  Support groups, education, community support |
| **3** | Support (“intervention terms”) | Intervention, incentive, program, scheme, policy, assistance, livelihood support, enabler |
| **4** | Impact (“outcome terms”) | Affect, effect, association, associated, consequence |
| **5** | Treatment (“outcome terms”) | Outcome, success, rates, unsuccessful, uptake, enrolment, adherence, cured, completed, treated, follow-up, loss to follow-up, relapse, recurrence, adverse outcome, diagnostic pathways, TB testing, quality of life, default, care cascade |
| **6** | Socioeconomic (“outcome terms”) | Outcome, financial burden, economic burden, economic consequences, social consequences, socioeconomic consequences, social impact, socioeconomic impact, costs, expenditure, expenses, spending, catastrophic expenditure, catastrophic costs, impoverishment, coping strategies, poverty, food security, loans, sold assets, dissaving, deprivation, defray, mitigate |
