# Supplementary material for: The impact of social protection interventions on treatment and socioeconomic outcomes of tuberculosis-affected people and households in low income, high burden settings: A systematic review and meta-analysis
Source: PLOS Glob Public Health. 2026 Sep 3;6(9):e0006704. doi: 10.1371/journal.pgph.0006704 (PMC13541142; doi:10.1371/journal.pgph.0006704)
Supplement: Supplementary file 6 [file pgph.0006704.s005.docx]

**S5 Text.** Search strategies

| # | Searches | Results |
| --- | --- | --- |
| 1 | (Tuberculosis[Title/Abstract] OR TB[Title/Abstract] OR "Mycobacterium tuberculosis" [Title/Abstract] OR "Pulmonary TB" [Title/Abstract] OR "TB-affected" [Title/Abstract] OR "TB infected" [Title/Abstract] OR "TB patients" [Title/Abstract] OR "Drug-resistant TB" [Title/Abstract] OR "TB individuals" [Title/Abstract] OR "TB-affected households" [Title/Abstract] OR "TB prevalent" [Title/Abstract] OR "Pulmonary tuberculosis" [Title/Abstract] OR "Pulmonary TB" [Title/Abstract] OR PTB[Title/Abstract]) AND (Social protection[Title/Abstract] OR "Social safety net" [Title/Abstract] OR "Socioeconomic support" [Title/Abstract] OR "Social support" [Title/Abstract] OR "Economic support" [Title/Abstract] OR "Financial support" [Title/Abstract]) AND ("2012"[Date - Publication] : "2021"[Date - Publication]) | 308 |
| 2 | (All TB treatment terms by title and abstract with OR as the Boolean operator) AND (All intervention terms by title and abstract with OR as the Boolean operator) AND ("2012"[Date - Publication] : "2021"[Date - Publication]) (i.e. #1 + additional terms) | 17,461 |
| 3 | (Tuberculosis[MeSH] + all TB treatment terms by title and abstract with OR as the Boolean operator) AND (All intervention terms by title and abstract with OR as the Boolean operator) AND ("2012"[Date - Publication] : "2021"[Date - Publication]) (i.e. #2+ MeSH terms) | 17,732 |
| 4 | ("2012"[Date - Publication] : "2021"[Date - Publication]) AND (Tuberculosis[MeSH] OR all TB treatment terms by title and abstract) AND (all intervention terms by title and abstract) AND (all outcome terms by title and abstract) (i.e. #3 + outcome terms). | 17,732 |

Web of Science search strategy

| # | Searches | Results |
| --- | --- | --- |
| 1 | TS=(TB terms with OR as the Boolean operator) AND TS=(all intervention terms with OR as the Boolean operator) AND TS=(all outcome terms with OR as the Boolean operator) | [28,985](https://apps-webofknowledge-com.ucsf.idm.oclc.org/summary.do?product=WOS&doc=1&qid=3&SID=8CXObna2otfnlRCFOFK&search_mode=AdvancedSearch&update_back2search_link_param=yes) |
| 2 | TI=(TB terms with OR as the Boolean operator) AND TS=(all intervention terms with OR as the Boolean operator) AND TS=(all outcome terms with OR as the Boolean operator) | [14,687](https://apps-webofknowledge-com.ucsf.idm.oclc.org/summary.do?product=WOS&doc=1&qid=4&SID=8CXObna2otfnlRCFOFK&search_mode=AdvancedSearch&update_back2search_link_param=yes) |
| 3 | TI=(TB terms with OR as the Boolean operator) AND TI=(all intervention terms with OR as the Boolean operator) AND TI=(all outcome terms with OR as the Boolean operator) | 1412 |
| 4 | TI=(TB terms with OR as the Boolean operator) AND TI=(all intervention terms with OR as the Boolean operator) AND TS=(all outcome terms with OR as the Boolean operator) | 3568 |
