# Supplementary material for: The impact of social protection interventions on treatment and socioeconomic outcomes of tuberculosis-affected people and households in low income, high burden settings: A systematic review and meta-analysis
Source: PLOS Glob Public Health. 2026 Sep 3;6(9):e0006704. doi: 10.1371/journal.pgph.0006704 (PMC13541142; doi:10.1371/journal.pgph.0006704)
Supplement: Supplementary file 8 [file pgph.0006704.s013.docx]

### **S2 Table.** Study characteristics of qualitative studies (n=4)

| **Authors** | **Study Title** | **Year of Publication** | **Country** | **Setting** |
| --- | --- | --- | --- | --- |
| George et al. | TB patient support systems in Kerala: A qualitative analysis. | 2021 | India | Urban |
| Kaliakbarova et al. | Psychosocial support improves treatment adherence among MDR-TB patients: Experience from East Kazakhstan | 2013 | Kazakhstan-an | Mixed, primarily urban |
| Orlandi et al. | Social incentives for adherence to tuberculosis treatment | 2019 | Brazil | Urban |
| Ukwaja et al. | Sustaining the DOTS': stakeholders' experience of a social protection intervention for TB in Nigeria | 2021 | Nigeria | Rural |
