# Supplementary material for: The impact of social protection interventions on treatment and socioeconomic outcomes of tuberculosis-affected people and households in low income, high burden settings: A systematic review and meta-analysis
Source: PLOS Glob Public Health. 2026 Sep 3;6(9):e0006704. doi: 10.1371/journal.pgph.0006704 (PMC13541142; doi:10.1371/journal.pgph.0006704)
Supplement: Supplementary file 9 [file pgph.0006704.s014.docx]

### **S3 Table.** Social protection interventions described in quantitative studies (n=51)

| **Study** | **Primary funding source** | **Social protection intervention type** | **Eligibility for social**  **protection intervention** | **TB sensitive vs. TB specific** |
| --- | --- | --- | --- | --- |
| Bhargava et al. | Study intervention/source | Nutritional support | Not described | Not described |
| Bhatt R et al. | Government/national TB program | Mixed with psychosocial support | Not described | Not described |
| Carter et al. | Government/national TB program | Cash | Not described | TB sensitive |
| Che et al. | Government/national TB program | Social health insurance | Not described | TB sensitive |
| Chenicer  et al. | Government/national TB program | Cash | Measures of poverty | TB sensitive |
| Ciobanu et al. | Mixed funding sources | Mixed without psychosocial support | TB status | TB sensitive |
| Dave et al. | Government/ national TB program | Cash | TB status | TB specific |
| de Souza et al. | Government/ national TB program | Cash | Measures of poverty | TB sensitive |
| Durovni et al. | Government/ national TB program | Cash | Measures of poverty | TB sensitive |
| Florentino et al. | Government/ national TB program | Mixed without psychosocial support | TB status | TB specific |
| Ismail et al. | Study intervention/source | Cash | TB status | TB specific |
| Jesus et al. | Government/ national TB program | Cash | Measures of poverty | TB sensitive |
| Jeyashree et al. | Government/national TB program | Cash | TB status | TB specific |
| Jiang et al. | Government/national TB program | Social health insurance | Not described | TB sensitive |
| Klein et al. | Government/national TB program | Cash | TB status | TB sensitive |
| Li et al. | Government/national TB program | Cash | TB status | TB sensitive |
| Liu et al. | Government | Social health insurance | Not described | TB sensitive |
| Lutge et al | Study intervention/source | Cash | Study intervention | TB specific |
| Mahapatra et al. | Study intervention/source | Nutritional support | Study intervention | TB specific |
| Mansour et al. | Government/ national TB program | Nutritional support | TB status | TB sensitive |
| Modi et al. | Government/ national TB program | Cash | TB status | TB specific |
| Ngamvithay-apong-Yanai et al. | Study intervention/source | Mixed with psychosocial support | Study intervention | TB specific |
| Oliosi et al. | Government/ national TB program | Cash | Measures of poverty | TB sensitive |
| Pavinati et al. | Government/national TB program | Cash | Not described | Not described |
| Pedrazzoli et al. | Government/ national TB program | Social health insurance | Not described | TB sensitive |
| Pham et al. | Government/ national TB program | Mixed without psychosocial support | TB status | TB speciific |
| Potty et al. | Study intervention/source | Psychosocial support | TB status | TB specific |
| Priedeman et al. | NGO/multilateral agency | Mixed without psychosocial support | TB status | TB sensitive |
| Randhawa et al. | Study intervention/source | Mixed with psychosocial support | TB status | TB specific |
| Reis-Santos et al (2022) | Study intervention/source | Nutritional support | Study intervention | TB specific |
| Reis-Santos et al. (2019) | Government/ national TB program | Cash | Measures of poverty | TB sensitive |
| Rogers et al. | NGO/multilateral agency | Mixed with psychosocial support | Study intervention | TB specific |
| Rohit et al. | Government/ national TB program | Cash | TB status | TB sensitive |
| Rudgard et al. | Government/ national TB program | Cash | Measures of poverty | TB sensitive |
| Samuel et al. | NGO/multilateral agency | Nutritional support | TB status | TB sensitive |
| Santos et al. | Government/ national TB program | Cash | Measures of poverty | TB sensitive |
| Singh et al. | Study intervention/source | Nutritional support | TB status | TB specific |
| Soares et al. | Government/national TB program | Primarily psychosocial support | TB status | TB sensitive |
| Sripad et al. | Government/national TB program | Cash | TB status | TB sensitive |
| Tian et al. | Government/national TB program | Social health insurance | Not described | TB sensitive |
| Timire et al. | Government/national TB program | Cash | TB status | TB specific |
| Torrens et al. | Government/national TB program | Cash | Measures of poverty | TB sensitive |
| Ukwaja et al. | Study intervention/source | Cash | TB status | TB sensitive |
| Wei et al. | Study intervention/source | Cash | Study intervention | TB specific |
| Wingfield et al. (2016a) | Study intervention/source | Mixed with psychosocial support | Study intervention | TB specific |
| Wingfield et al. (2016b) | Study intervention/source | Mixed with psychosocial support | Study intervention | TB specific |
| Wingfield et al. (2017) | Study intervention/source | Mixed with psychosocial support | Study intervention | TB specific |
| Wrohan et al. | Government/national TB program | Social health insurance | Not described | TB sensitive |
| Xiang et al. | Government/national TB program | Cash | Other | TB specific |
| Yin et al. | Not specified | Mixed with psychosocial support | Study intervention | TB specific |
| Zhao et al. | NGO/multilateral agency | Cash | TB status | TB specific |
