# Supplementary material for: The impact of social protection interventions on treatment and socioeconomic outcomes of tuberculosis-affected people and households in low income, high burden settings: A systematic review and meta-analysis
Source: PLOS Glob Public Health. 2026 Sep 3;6(9):e0006704. doi: 10.1371/journal.pgph.0006704 (PMC13541142; doi:10.1371/journal.pgph.0006704)
Supplement: Supplementary file 10 [file pgph.0006704.s015.docx]

### **S4 Table.** Outcomes and implementation challenges described in quantitative studies (n=46). “Mix of other TB treatment outcomes” include mortality, TB treatment default (treatment interruption of at least two months), loss to follow up, or treatment failure.

| **Study** | **Primary outcome** | **Secondary outcome** | **Primary implementation challenge** | **Secondary implementation challenge** |
| --- | --- | --- | --- | --- |
| Bhargava et al. | TB treatment success | Mix of other TB treatment outcomes | Not described | Not described |
| Bhatt R et al. | TB treatment success | Mix of other TB treatment outcomes | Not described | Not described |
| Carter et al. | TB treatment success | N/A | Not described | Not described |
| Che et al. | Unfavorable treatment outcomes | N/A | Not described | Not described |
| Chenicer et al. | Unfavorable treatment outcomes | Mix of other TB treatment outcomes | Not described | Not described |
| Ciobanu et al. | TB treatment success | Mix of other TB treatment outcomes | Not described | Not described |
| Dave et al. | Unfavorable treatment outcomes | TB treatment success | Delayed payments or non-receipt of payment | Insufficient funds |
| de Souza et al. | Death | N/A | Not described | Not described |
| Durovni et al. | TB treatment success | N/A | Not described | Not described |
| Florentino et al. | Catastrophic costs and dissaving | Financial coping mechanisms | Not described | Not described |
| Ismail et al | TB treatment success | Mix of other TB treatment outcomes | Not described | Not described |
| Jesus et al | TB incidence and mortality | Case fatality rate | Not described | Not described |
| Jeyashree et al. | TB treatment success | Mix of other TB treatment outcomes | Delayed payments or non-receipt of payment | Not described |
| Jiang et al. | Loss to follow up | N/A | Not described | Not described |
| Klein et al. | TB treatment success | Mix of other TB treatment outcomes | Administrative issues (inadequate banking systems/issues with paperwork or forms required) | Lack of knowledge/awareness about the program or how to link patients to the program (patient or provider/provisioner) |
| Li et al. | Other positive TB treatment outcomes | Socioeconomic outcome | Not described | Not described |
| Liu et al. | TB treatment success | Out of pocket expenses | Not described | Not described |
| Lutge et al. | TB treatment success | N/A | Administrative issues (inadequate banking systems/issues with paperwork or forms required, delayed disbursement or non-receipt of funds/benefit) | Not described |
| Mahapatra et al. | TB treatment success | Mix of other TB treatment outcomes | Not described | Not described |
| Mansour et al. | Loss to follow up | N/A | Not described | Not described |
| Modi et al. | TB treatment success | Mix of other TB treatment outcomes | Administrative issues (inadequate banking systems/issues with paperwork or forms required, delayed disbursement or non-receipt of funds/benefit) | Lack of knowledge/awareness about the program or how to link patients to the program (patient or provider/provisioner) |
| Ngamvithay-apong-Yanai et al. | TB treatment success | Treatment failure | Not described | Not described |
| Oliosi et al. | TB treatment success | Mix of other TB treatment outcomes | Not described | Not described |
| Pavinati et al. | Unfavorable treatment outcomes | Cure | Not described | Not described |
| Pedrazzoli et al. | Catastrophic costs | Direct and indirect costs | Not described | Not described |
| Pham et al. | Catastrophic costs | Household income, income loss | Not described | Not described |
| Potty et al. | TB treatment success | N/A | Not described | Not described |
| Priedeman et al. | TB treatment success | Mix of other TB treatment outcomes | Not described | Not described |
| Randhawa et al. | TB treatment success | Mix of other TB treatment outcomes | Not described | Not described |
| Reis-Santos et al. (2022) | TB treatment success | Nutritional support | Not described | Not described |
| Reis-Santos et al. (2019) | TB treatment success | N/A | Not described | Not described |
| Rogers et al. | TB treatment success | Mix of other TB treatment outcomes | Not described | Not described |
| Rohit et al. | TB treatment success | Mix of other TB treatment outcomes | Administrative issues (inadequate banking systems/issues with paperwork or forms required, delayed disbursement or non-receipt of funds/benefit) | Lack of knowledge/awareness about the program or how to link patients to the program (patient or provider/provisioner) |
| Rudgard et al | Socioeconomic outcome | N/A | Not described | Not described |
| Samuel et al. | Unfavorable treatment outcome | Mix of other TB treatment outcomes | Not described | Not described |
| Santos et al. | Unfavorable TB treatment outcomes | Mix of other TB treatment outcomes | Not described | Not described |
| Singh et al. | Treatment success | Weight gain | Not described | Not described |
| Soares et al. | TB treatment success | Mix of other TB treatment outcomes | Not described | Not described |
| Sripad et al. | Treatment default | N/A | Administrative issues (inadequate banking systems/issues with paperwork or forms required) | Not described |
| Tian et al. | TB treatment success | Mix of other TB treatment outcomes | Not described | Not described |
| Timire et al. | TB treatment success | Mix of other TB treatment outcomes | Administrative issues (inadequate banking systems/issues with paperwork or forms required, delayed disbursement or non-receipt of funds/benefit), insufficient funds | Lack of knowledge/awareness about the program or how to link patients to the program (patient or provider/provisioner) |
| Torrens et al. | TB treatment success | N/A | Not described | Not described |
| Ukwaja et al | TB treatment success | Mix of other TB treatment outcomes | Not described | Not described |
| Wei et al. | TB treatment success | Mix of other TB treatment outcomes | Administrative issues (inadequate banking systems/issues with paperwork or forms required) | Not described |
| Wingfield et al. (2016a) | Socioeconomic outcome | N/A | Not described | Not described |
| Wingfield et al. (2016b) | Socioeconomic outcome | N/A | Not described | Not described |
| Wingfield et al. (2017) | TB treatment success | Mix of other TB treatment outcomes | Not described | Not described |
| Wrohan et al | Loss to follow up | TB treatment success | Not described | Not described |
| Xiang et al. | Socioeconomic outcome | N/A | Not described | Not described |
| Yin et al. | TB treatment success | Mix of other TB treatment outcomes | Not described | Not described |
| Zhao et al. | Socioeconomic outcome | N/A | Other-insufficient funds | Not described |
