# Supplementary material for: The impact of social protection interventions on treatment and socioeconomic outcomes of tuberculosis-affected people and households in low income, high burden settings: A systematic review and meta-analysis
Source: PLOS Glob Public Health. 2026 Sep 3;6(9):e0006704. doi: 10.1371/journal.pgph.0006704 (PMC13541142; doi:10.1371/journal.pgph.0006704)
Supplement: Supplementary file 11 [file pgph.0006704.s016.docx]

### **S5 Table.** Risk of bias assessments of non-randomized studies using the Newcastle Ottawa Scale (NOS).

| **Study** | **Selection (maximum score=4)** | **Comparability**  **(maximum score=2)** | **Outcome (maximum score=3)** |
| --- | --- | --- | --- |
| Bhargava, 2023 | 4 | 2 | 3 |
| Bhatt et al., 2019 | 3 | 0 | 3 |
| Carter et al., 2019 | 4 | 2 | 3 |
| Che et al., 2025 | 4 | 2 | 3 |
| Chenicer et al., 2021 | 4 | 2 | 3 |
| Ciobanu et al., 2014 | 4 | 2 | 3 |
| Dave et al., 2022 | 4 | 2 | 3 |
| de Souza et al., 2018 | 4 | 2 | 3 |
| Durovni et al., 2018 | 4 | 2 | 3 |
| Florentino et al. 2022 | 4 | 2 | 3 |
| Jesus et al 2025 | 4 | 2 | 2 |
| Jeyashree et al., 2024 | 4 | 2 | 3 |
| Jiang et al. 2023 | 4 | 2 | 3 |
| Klein et al., 2019 | 4 | 2 | 3 |
| Li et al., 2015 | 4 | 2 | 3 |
| Liu et al., 2023 | 4 | 2 | 3 |
| Mahapatra et al. | 4 | 2 | 3 |
| Mansour et al., 2018 | 4 | 2 | 3 |
| Modi et al., 2020 | 3 | 0 | 2 |
| Ngamvithayapong-Yanai et al., 2013 | 3 | 1 | 3 |
| Oliosi et al., 2019 | 4 | 2 | 3 |
| Pavinati et al., 2024 | 4 | 2 | 2 |
| Pedrazzoli et al., 2021 | 4 | 2 | 3 |
| Pham et al., 2023 | 4 | 2 | 3 |
| Potty et al., 2023 | 4 | 2 | 3 |
| Priedeman et al., 2018 | 4 | 2 | 3 |
| Randhawa et al., 2023 | 1 | 0 | 1 |
| Reis-Santos et al., 2019 | 4 | 2 | 3 |
| Rogers et al., 2018 | 4 | 2 | 3 |
| Rohit et al., 2020 | 4 | 2 | 3 |
| Rudgard et al., 2018 | 4 | 2 | 3 |
| Samuel et al., 2016 | 4 | 1 | 3 |
| Santos et al | 4 | 2 | 2 |
| Singh et al., 2021 | 4 | 1 | 3 |
| Soares et al., 2013 | 4 | 2 | 3 |
| Sripad et al., 2014 | 4 | 2 | 3 |
| Tian et al | 4 | 2 | 3 |
| Timire et al., 2022 | 4 | 2 | 3 |
| Torrens et al., 2016 | 4 | 2 | 3 |
| Ukwaja et al., 2017 | 4 | 2 | 3 |
| Wei et al., 2012 | 4 | 2 | 3 |
| Wingfield et al., 2026 | 4 | 2 | 3 |
| Wrohan et al., 2022 | 4 | 2 | 3 |
| Xiang et al., 2016 | 4 | 2 | 3 |
| Yin et al., 2018 | 1 | 0 | 0 |
| Zhao et al., 2013 | 4 | 2 | 3 |
