# Supplementary material for: The impact of social protection interventions on treatment and socioeconomic outcomes of tuberculosis-affected people and households in low income, high burden settings: A systematic review and meta-analysis
Source: PLOS Glob Public Health. 2026 Sep 3;6(9):e0006704. doi: 10.1371/journal.pgph.0006704 (PMC13541142; doi:10.1371/journal.pgph.0006704)
Supplement: Supplementary file 12 [file pgph.0006704.s017.docx]

### **S6 Table.** Risk of bias assessments of randomized controlled trials (RCTs) using the Cochrane Risk of Bias (RoB) assessment tool.

| **Study** | **Deviation from intervention** | **Missing outcome data** | **Outcome measurement** | **Selection of reported result** | **Overall risk assessment** |
| --- | --- | --- | --- | --- | --- |
| Ismail et al., 2025 | Some concern | Some concern | Low risk of bias | Low risk of bias | Low risk |
| Lutge et al., 2013 | Some concern | Low risk of bias | Low risk of bias | Low risk of bias | Low risk |
| Reis-Santos et al. 2022 | Low risk of bias | Some concern | Low risk of bias | Low risk of bias | Low risk |
| Wingfield et al., 2016 | Low risk of bias | Low risk of bias | Low risk of bias | Low risk of bias | Low risk |
| Wingfield et al., 2017 | Low risk of bias | Low risk of bias | Low risk of bias | Low risk of bias | Low risk |
