# Supplementary material for: The impact of social protection interventions on treatment and socioeconomic outcomes of tuberculosis-affected people and households in low income, high burden settings: A systematic review and meta-analysis
Source: PLOS Glob Public Health. 2026 Sep 3;6(9):e0006704. doi: 10.1371/journal.pgph.0006704 (PMC13541142; doi:10.1371/journal.pgph.0006704)
Supplement: Supplementary file 13 [file pgph.0006704.s018.docx]

**S7 Table.** Studies that reported a primary outcome of TB treatment success that were excluded from the meta-analysis.

| **Study** | **Intervention type** | **Rationale for exclusion from meta-analysis** |
| --- | --- | --- |
| Bhargava et al. | Nutritional support | Did not include a control group |
| Checiner et al. | Cash | Data reported in studies not able to be extracted for meta-analysis calculations |
| Dave et al. | Cash | Data reported in studies not able to be extracted for meta-analysis calculations |
| Liu et al. | Social health insurance | Did not include a control group |
| Randhawa et al. | Mixed with psychosocial support | Could not compare historical (control) cohort to intervention cohort |
| Tian et al. | Social health insurance | Data reported in studies not able to be extracted for meta-analysis calculations |
| Wrohan et al. | Social health insurance | Did not include sufficient details of the cohort of individuals not using social health insurance |
