# Supplementary material for: The impact of social protection interventions on treatment and socioeconomic outcomes of tuberculosis-affected people and households in low income, high burden settings: A systematic review and meta-analysis
Source: PLOS Glob Public Health. 2026 Sep 3;6(9):e0006704. doi: 10.1371/journal.pgph.0006704 (PMC13541142; doi:10.1371/journal.pgph.0006704)
Supplement: Supplementary file 14 [file pgph.0006704.s019.docx]

**S8 Table.** A listing of all studies, both included and excluded, for reasons for exclusion if applicable.

| **Number** | **Authors and date** | **Study Title** | **Eligible for inclusion** | **Reason for exclusion** |
| --- | --- | --- | --- | --- |
| 1 | Aderno et al., 2012 | Support and social care of tuberculosis in pediatrics | No | Ineligible study design |
| 2 | [Aragão](https://pubmed.ncbi.nlm.nih.gov/?term=Arag%C3%A3o+FBA&cauthor_id=38896660) et al.,  2024 | Social protection in areas vulnerable to tuberculosis: a mixed methods study in São Luís, Maranhão | No | Ineligible outcomes |
| 3 | Amon et al., 2022 | The impact of scaling up human rights interventions on reducing inequality and increasing access to care and treatment for HIV and TB: Mid-term results from the Breaking Down Barriers initiative | No | Ineligible study design |
| 4 | Balakrishnan et al., 2015 | Social inclusion: An effort to end loss-to-treatment follow-up in tuberculosis | No | Ineligible intervention |
| 5 | Begum et al. | Utilisation of nutritional support scheme among the patients of tuberculosis: A myth or a truth | No | Ineligible outcome |
| 6 | Bhargava  et al., 2023 | Nutritional support for adult patients with microbiologically confirmed pulmonary tuberculosis: outcomes in a programmatic cohort nested within the RATIONS trial in Jharkland, India | Yes | N/A |
| 7 | Bhatt R  et al., 2019 | Impact of integrated psycho-socio-economic support on treatment outcome in drug resistant tuberculosis - A retrospective cohort study | Yes | N/A |
| 8 | Carter  et al., 2019 | The impact of a cash transfer programme on tuberculosis treatment success rate: a quasi-experimental study in Brazil. | Yes | N/A |
| 9 | Cassidy et al., 2018 | Food Insecurity and Tuberculosis: Policy Urgently Needs To Play Catch-Up | No | Ineligible outcome |
| 10 | Cavalcanti et al. 2023 | Evaluation and Forecasting Analysis of the Association of Conditional Cash Transfer With Child Mortality in Latin America, 2000-2030 | No | Ineligible time frame |
| 11 | Che et al., 2025 | Attrition in the treatment cascade among elderly patients with multidrug-resistant TB |  | N/A |
| 12 | Chen et al., 2024 | Impact of financial support on treatment outcomes of multidrug-resistant tuberculosis: A population-based, retrospective cohort study in Shanghai, China | No | Ineligible time frame |
| 13 | Chenicer  et al., 2021 | Social and health factors associated with unfavourable treatment outcome in adolescents and young adults with tuberculosis in Brazil: a national retrospective cohort study | Yes | N/A |
| 14 | Ciobanu  et al., 2014 | Do incentives improve tuberculosis treatment outcomes in the Republic of Moldova? | Yes | N/A |
| 15 | Claros et al 2014 | Adherence to HIV and TB care and treatment, the role of food security and nutrition | No | Ineligible outcome |
| 16 | Cocozza et al. 2020 | An assessment of current tuberculosis patient care and support policies in high-burden countries | No | Ineligible outcome |
| 17 | Contreras et al., 2017 | Addressing tuberculosis patients' medical and socio-economic needs: a comprehensive programmatic approach | No | Ineligible outcome |
| 18 | Dave et al. 2022 | Does Direct Benefit Transfer Improve Outcomes Among People With Tuberculosis? – A Mixed-Methods Study on the Need for a Review of the Cash Transfer Policy in India | Yes | N/A |
| 19 | dePaulaMartins et al. 2019 | Effect of the Bolsa Familiia Programme on tuberculosis treatment outcomes | No | Ineligible study design |
| 20 | de Souza  et al. 2018 | Family health and conditional cash transfer in Brazil and its effect on tuberculosis mortality | Yes | N/A |
| 21 | Deshmukh et al. 2018 | Social support a key factor for adherence to multidrug-resistant tuberculosis treatment | No | Ineligible outcome |
| 22 | Diaw et al., 2018 | Implementing TB control in a rural, resource-limited setting: the stop-TB Italia project in Senegal | No | Ineligible intervention |
| 23 | Du et al., 2024 | Health insurance's contribution to reducing the financial burden of tuberculosis in Guizhou Province, China. | No | Ineligible population |
| 24 | Durovni  et al., 2018 | The impact of the Brazilian family health strategy and the conditional cash transfer on tuberculosis treatment outcomes in Rio De Janeiro: an individual-level analysis of secondary data | Yes | N/A |
| 25 | Elias et al., 2015 | Material benefits as an incentive to reduce abandonment of treatment of tuberculosis in people living in street: review of economic analysis | Yes | Ineligible outcome |
| 26 | Florentino et al. 2022 | Expansion of social protection is necessary towards zero catastrophic costs due to TB: The first national TB patient cost survey in the Philippines | Yes | N/A |
| 27 | Hu et al., 2021 | Evaluation of the effect of the capitation compensation mechanism among pulmonary tuberculosis patients with a full period of treatment | No | Ineligible outcome |
| 28 | Inthavong et al., 2025 | Does nutritional support contribute to mitigating the financial burden faced by TB-affected households? | No | Ineligible intervention |
| 29 | Ismail et al., 2025 | Effects of conditional cash transfers and pre-test and post-test tuberculosis counselling on patient outcomes and loss to follow-up across the continuum of care in South Africa: a randomised controlled trial | Yes | N/A |
| 30 | Jesus et al., 2025 | Effects of conditional cash transfers on tuberculosis incidence and mortality according to race, ethnicity and socioeconomic factors in the 100 Million Brazilian Cohort | Yes | N/A |
| 31 | Jeyashree et al., 2024 | Ni-kshay Poshan Yojana: receipt and utilization among persons with TB notified under the National TB Elimination Program in India, 2022 | Yes | N/A |
| 32 | Jiang et al., 2023 | Factors associated with loss to follow-up before and after treatment initiation among patients with tuberculosis: A 5-year observation in China | Yes | N/A |
| 33 | Jiang et al.,  2025 | Differences Between the Local and Migrant Populations in Healthcare Service Use and Direct Cost of Tuberculosis Treatment - Shanghai Municipality, China, 2020-2021 | Noo | Ineligible population |
| 34 | Kim et al., 2018 | Tuberculosis Relief Belt Supporting Project (Tuberculosis Patient Management Project for Poverty Group) | No | Ineligible intervention |
| 35 | Klein et al., 2019 | Correction: Evaluation of a social protection policy on tuberculosis treatment outcomes: A prospective cohort study | No | Ineligible study design |
| 36 | Klein et al., 2019 | Evaluation of a social protection policy on tuberculosis treatment outcomes: A prospective cohort study | Yes | N/A |
| 37 | Kliner et al., 2015 | Effects of financial incentives for treatment supporters on tuberculosis treatment outcomes in Swaziland: a pragmatic interventional study | No | Ineligible population |
| 38 | Kumar et al., 2020 | Nikshay Poshan Yojana (NPY) for tuberculosis patients: Early implementation challenges in Delhi, India | No | Ineligible outcome |
| 39 | Lee et al., 2019 | Incentives that influence low income Filipinos with Tuberculosis symptoms to change health-seeking behaviour: a randomized controlled trial | No | Ineligible outcome |
| 40 | Li et al., 2015 | Effect of a comprehensive programme to provide universal access to care for sputum-smear-positive multidrug-resistant tuberculosis in China: a before-and-after study. | Yes | N/A |
| 41 | Liu et al., 2023 | Impacts of Medical Security Level on Treatment Outcomes of Drug-Resistant Tuberculosis: Evidence from Wuhan City, China | Yes | N/A |
| 42 | Lönnroth et al., 2014 | Beyond UHC: monitoring health and social protection coverage in the context of tuberculosis care and prevention. | No | Ineligible study design |
| 43 | Lutge et al., 2013 | Economic support to improve tuberculosis treatment outcomes in South Africa: a pragmatic cluster-randomized controlled trial. | Yes | N/A |
| 44 | Mahapatra et al., 2024 | Effectiveness of food supplement on treatment outcomes and quality of life in pulmonary tuberculosis: Phased implementation approach | Yes | N/A |
| 45 | Malacarne et al., 2018 | Factors associated with TB in an indigenous population in Brazil: the effect of a cash transfer program | No | Ineligible intervention |
| 46 | Mansour  et al., 2018 | Impact of a nutritional support programme on loss to follow-up after tuberculosis diagnosis in Kenya | Yes | N/A |
| 47 | Modi  et al., 2020 | Financial Incentive - does this have an impact on outcome of Tuberculosis? | Yes | N/A |
| 48 | Nery et al., 2017 | Effect of Brazil's conditional cash transfer programme on tuberculosis incidence | No | Ineligible outcome |
| 49 | Nidoi et al., 2021 | Impact of socio-economic factors on Tuberculosis treatment outcomes in north-eastern Uganda: a mixed methods study | No | Ineligible intervention |
| 50 | Ngamvithay apong-Yanai et al., 2013 | Engaging women volunteers of high socioeconomic status in supporting socioeconomically disadvantaged tuberculosis patients in Chiang Rai, Thailand. | Yes | N/A |
| 51 | Oliosi et al., 2019 | Effect of the Bolsa Familia Programme on the outcome of tuberculosis treatment: a prospective cohort study. | Yes | N/A |
| 52 | Parthasarathy et al., 2019 | Cash transfer interventions in tuberculosis treatment outcomes | No | Ineligible study design |
| 53 | Patel et al., 2019 | Cash transfer scheme for people with tuberculosis treated by the National TB Programme in Western India: a mixed methods study | No | Ineligible outcomes |
| 54 | Pavinati et al., 2024 | Vulnerability to loss of follow-up and death due to tuberculosis among homeless individuals in Brazil: a retrospective cohort study | Yes | N/A |
| 55 | Pedrazzoli et al., 2021 | Does Ghana’s National Health Insurance Scheme provide financial protection to tuberculosis patients and their households? | Yes | N/A |
| 56 | Pham et al., 2023 | Determinants of catastrophic costs among households affected by multi-drug resistant tuberculosis in Ho Chi Minh City, Viet Nam: a prospective cohort study | Yes | N/A |
| 57 | Potty et al., 2023 | Tuberculosis treatment outcomes and patient support groups, southern India | Yes | N/A |
| 58 | Priedeman et al., 2018 | Evaluating the impact of social support services on tuberculosis treatment default in Ukraine | Yes | N/A |
| 59 | Randhawa et al., 2023 | Outcome Optimization for Patients with Drug-Resistant TB Via The Implementation of An All-Inclusive Care Program | Yes | N/A |
| 60 | Reis-Santos et al., 2022 | A Matter of Inclusion: A Cluster-Randomized Trial to Access the Effect of Food Vouchers Versus Traditional Treatment on Tuberculosis Outcomes in Brazil | Yes | N/A |
| 61 | Reis-Santos et al., 2019 | Tuberculosis in Brazil and cash transfer programs: A longitudinal database study of the effect of cash transfer on cure rates. | Yes | N/A |
| 62 | Rogers  et al., 2018 | Impact of community-based adherence support on treatment outcomes for tuberculosis, leprosy and HIV/AIDS-infected individuals in post-Ebola Liberia. | Yes | N/A |
| 63 | Rohit et al., 2020 | Does provision of cash incentive to HIV-infected tuberculosis patients improve the treatment success in programme settings? A cohort study from South India. | Yes | N/A |
| 64 | Rudgard  et al. 2017 | Uptake of governmental social protection and financial hardship during drug-resistant tuberculosis treatment in Rio de Janeiro, Brazil. | Yes | N/A |
| 65 | Samuel  et al., 2015 | Relationship between nutritional support and tuberculosis treatment outcomes in West Bengal India | Yes | N/A |
| 66 | Santos et al., 2024 | Social and health factors associated with unfavourable treatment outcomes in children and adolescents with drug-sensitive tuberculosis in Brazil: a national retrospective cohort study | Yes | N/A |
| 67 | Shah et al., 2023 | Notes for Challenges and Strategic Solutions to Guarantee Last Mile Reach for an Indian TB Patient’s Nikshay Poshan Yojana; A Conditional Cash Transfer Scheme comment on “Does Direct Benefit Transfer Improve Outcomes Among People With Tuberculosiis? A Mixed-Method Study on the Need for a Review of the Cash Transfer Policy in India | No | Ineligible outcomes |
| 68 | Shat et al., 2023 | Challenges and Strategic Solutions to Guarantee Last Mile Reach for an Indian TB Patient’s Nikshay Poshan Yojana; A Conditional Cash Transfer Scheme; Comment on “Does Direct Benefit Transfer Improve Outcomes Among People With Tuberculosis? – A Mixed-Methods Study on the Need for a Review of the Cash Transfer Policy in India” | No | Ineligible study design |
| 69 | Shah et al., 2024 | Nutritional Supplementation in Tuberculosis Treatment: A Mixed Methods Study of Clinical Outcomes and Patient Perceptions in Jamnagar, India. | No | Ineligible intervention |
| 70 | Singh et al., 2021 | Improving tuberculosis treatment success rate through nutrition supplements and counseling: Findings from a pilot intervention in India | Yes | N/A |
| 71 | Siroka et al., 2016 | Association between spending on social protection and tuberculosis burden: a global analysis | No | Ineligible outcome |
| 72 | Soares et al., 2013 | Tuberculosis control in a socially vulnerable area: a community intervention beyond DOT in a Brazilian favela. | Yes | N/A |
| 73 | Sripad et al., 2014 | Effects of Ecuador's national monetary incentive program on adherence to treatment for drug-resistant tuberculosis. | Yes | N/A |
| 74 | Suwannakeeree et al., 2015 | A Medication Adherence Enhancement Program for Persons with Pulmonary Tuberculosis: A Randomized Controlled Trial Study. Pacific Rim International Journal of Nursing Research | No | Ineligible intervention |
| 75 | Testov et al., 2014 | Impact of social support programme on MDR-TB patients' treatment outcomes | No | Ineligible study design |
| 76 | Tian et al., 2025 | Determinants of treatment success and cost implications in MDR/RR-TB patients: a prospective cohort study in China | Yes | N/A |
| 77 | Timire et al. 2022 | Coverage and effectiveness of conditional cash transfer for people with drug resistant tuberculosis in Zimbabwe: A mixed methods study | Yes | N/A |
| 78 | Torrens  et al., 2016 | Effectiveness of a conditional cash transfer programme on TB cure rate: A retrospective cohort study in Brazil | Yes | N/A |
| 79 | Traore et al., 2022 | The high costs facing TB-affected households in Mali | No | Ineligible intervention |
| 80 | Ukwaja  et al., 2017 | Economic Support Intervention improves tuberculosis treatment outcomes in Rural Nigeria | Yes | N/A |
| 81 | Vanleeuw et al., 2022 | I’m suffering for food”: Food insecurity and access to social protection for TB patients and their households in Cape Town, South Africa | No | Ineligible outcome |
| 82 | Wei et al., 2012 | Providing financial incentives to rural-to-urban tuberculosis migrants in Shanghai: an intervention study. | Yes | N/A |
| 83 | Wei et al., 2015 | Effective reimbursement rates of the rural health insurance among uncomplicated tuberculosis patients in China | No | Ineligible intervention |
| 84 | Wingfield et al., 2015 | Designing and implementing a socioeconomic intervention to enhance TB control: operational evidence from the CRESIPT project in Peru. BMC Public Health | No | Ineligible study design |
| 85 | Wingfield et al., 2016 | Beyond pills and tests: addressing the social determinants of tuberculosis | Yes | N/A |
| 86 | Wingfield et al., 2016 | The economic effects of supporting tuberculosis-affected households in Peru. | Yes | N/A |
| 87 | Wingfield et al., 2017 | A randomized controlled study of socioeconomic support to enhance tuberculosis prevention and treatment, Peru. | Yes | N/A |
| 88 | Wrohan  et al., 2022 | Predictors of treatment outcomes among patients with multidrug-resistant tuberculosis in Vietnam: a retrospective cohort study | Yes | N/A |
| 89 | Xiang et al., 2016 | The impact of the new cooperative medical scheme on financial burden of tuberculosis patients: evidence from six counties in China. | Yes | N/A |
| 90 | Yin et al., 2018 | The relationship between social support, treatment interruption and treatment outcome in patients with multidrug resistant tuberculosis in China: A mixed methods study. | Yes | N/A |
| 91 | Zhao et al., 2013 | Impacts of the "transport subsidy initiative on poor TB patients" in Rural China: a patient-cohort based longitudinal study in rural china | Yes | N/A |
