# Supplementary material for: The impact of social protection interventions on treatment and socioeconomic outcomes of tuberculosis-affected people and households in low income, high burden settings: A systematic review and meta-analysis
Source: PLOS Glob Public Health. 2026 Sep 3;6(9):e0006704. doi: 10.1371/journal.pgph.0006704 (PMC13541142; doi:10.1371/journal.pgph.0006704)
Supplement: Supplementary file 15 [file pgph.0006704.s020.docx]

**S9 Table.** GRADE assessment of certainty of evidence.

| **Outcome** | **Number of participant (studies)** | **Effect (meta-analysis)** | **Risk of bias** | **Inconsistency** | **Indirectness** | **Imprecision** | **Publication bias** | **Overall certainty (GRADE)** | **Comments** |
| --- | --- | --- | --- | --- | --- | --- | --- | --- | --- |
| TB treatment success | 190,493 (26 studies, 3 of which were RCTs) | 2.15 | Low* | Not serious** | Not serious*** | Not serious** | Undetected | High | Distribution of study effects is relatively narrow, with almost all studies reporting a positive effect |

*Refer to S2, Tables 5 and 6 for complete risk of bias assessments

**The distribution of study effects is relatively narrow, with almost all studies reporting a positive effect. Our pooled estimate had a confidence interval of 1.76 to 2.64, suggesting moderate heterogeneity

***Studies included directly addressed our PICOT question
