# Supplementary material for: The impact of social protection interventions on treatment and socioeconomic outcomes of tuberculosis-affected people and households in low income, high burden settings: A systematic review and meta-analysis
Source: PLOS Glob Public Health. 2026 Sep 3;6(9):e0006704. doi: 10.1371/journal.pgph.0006704 (PMC13541142; doi:10.1371/journal.pgph.0006704)
Supplement: Supplementary file 18 [file pgph.0006704.s025.docx]

Dear Editor and Reviewers,

We thank you sincerely for taking the time to review our prior submission of this manuscript (**PGPH-D-25-03753**). We appreciate the thoughtful comments, and the feedback that will improve the quality and rigor of our submission. We have addressed these comments and are re-submitting as a new submission accordingly, at the instruction of Dr. Deepavali A. Dhond at PLOS Global Public Health.

The editor noted the following concerns in our prior submission:

1. *In the case of your submission, we are concerned that the literature review was completed over a year ago. Systematic and scoping reviews should provide an up-to-date overview of the research topic covered, and we do not feel that this study meets this requirement.*

In our prior version, we last updated our search January 2025. We subsequently updated our search in December 2025 and included eligible articles published by December 2025.

2. *Additionally, we note that language restrictions are imposed on the literature searches without adequate justification. There is increasing recognition that exclusion of studies published in other languages can increase the risk of bias in systematic reviews. We feel that any restrictions should be justified based on scientific reasons rather than resource constraints. As a result, we have concerns about the comprehensiveness of the review and the validity of the conclusions*

We have clarified that our search did not restrict by language. Although the final set of eligible articles only included articles written in English, we did not restrict by language when conducting our search. We have updated our manuscript accordingly.

We hope that this revised manuscript meets your expectations and addresses the concerns that the editor raised. We would be very grateful if this manuscript could be considered for publication in PLOS Global Public Health.

Sincerely,

Mollie Hudson
