# Supplementary material for: The impact of social protection interventions on treatment and socioeconomic outcomes of tuberculosis-affected people and households in low income, high burden settings: A systematic review and meta-analysis
Source: PLOS Glob Public Health. 2026 Sep 3;6(9):e0006704. doi: 10.1371/journal.pgph.0006704 (PMC13541142; doi:10.1371/journal.pgph.0006704)
Supplement: Supplementary file 19 [file pgph.0006704.s026.docx]

**Modifications to the manuscript as requested by the editor:**

As requested, per the data availability policy, we have now included the following:

1. A numbered table of all studies identified in the literature search, including those that were excluded from the analyses. For every excluded study, we have included the reason(s) for exclusion. Please note that there were no unpublished studies included in the analysis (S8, Table 1).
2. Additionally, please note that data or supporting information were not obtained from another source (e.g. correspondence with the author of the original research article).
3. We have included a table of all data extracted from the primary research sources for the systematic review and meta-analysis, including the data extractors, date of data extraction, and confirmation that the study was eligible to be included in the review (S6, Table 1).
4. We have included a table of all data extracted from each study for the reported meta-analysis that would be required to replicate our analyses (S7, Table 1).
5. We have removed our supplementary materials from the manuscript file as requested, and replaced with the file type “Supporting Information.” We have also now included a list of legends immediately after the reference list, as requested.
6. Lastly, of note, the authors realized that we had miscounted the number of excluded studies. The PRISMA flow chat has been modified accordingly.

**Responses to reviewers:**

First and foremost, we are very grateful to the reviewers and thank them for their insightful and constructive comments, which have helped to strengthen then manuscript to make it suitable for publication in PLOS Global Public Health. Below, we have addressed each point made by both reviewers.

1. Both reviewers commented in the high heterogeneity of our meta-analysis, and that this is insufficiently explored or interpreted. First, we thank the reviewers for their attention to detail and thoughtful responses, and agree that the heterogeneity needed to be more substantively addressed. We also note and appreciate the between-study heterogeneity evident from our meta-analysis relates to a concern raised by Reviewer 2 that using the World Bank’s definition of social protection, which is inherently more inclusive, introduces a significant challenge of describing and analyzing a wide variety of social protection interventions. We have aimed to address these concerns by conducting the following careful and comprehensive, additional analyses:
   - Meta-regression (which was included previously but now, as suggested by Reviewer 1, is included with relevant citations to clarify the rationale for variable selection) (page 13, lines 326-327).
   - The addition and interpretation of a prediction interval (S5, Figure 1)
   - Leave-one-out analysis to determine if a single outlier study was driving heterogeneity, as well as an interpretation of results (S5, Figure 2)
   - To address a specific concern of Reviewer 2, we have now conducted subgroup analysis disaggregating TB specific and TB sensitive social protection interventions, with accompanying additions to both the results (page 14, lines 406-409), discussion (pages 18-19, lines 339-356), and supplementary file (S5, Figures 3-6).
   - To address Reviewer 1’s concerns regarding the overrepresentation of middle-income settings, we have now conducted subgroup analysis by WHO region (S5, Figure 5).

Additionally, we added the following to the methods section to clarify types of studies that would not be included (page 7, lines 173-175):

*“We excluded interventions that were not intended to reduce socioeconomic risk; for example, nutritional support as a clinical intervention during a hospitalization was not considered to be social protection, and studies describing type of intervention were not included.”*

2. We appreciate Reviewer 1’s comment that “The conclusion that social protection “significantly improves” outcomes may be overstated given heterogeneity and predominance of observational studies.” We have modified our text accordingly, and have instead stated (page 18-19, lines 457-461):

*“In conclusion, social protection interventions have the potential to significantly improve TB treatment outcomes. Our meta-analysis and subgroup analysis indicate that, on average, individuals who had access to social protection interventions had twice the odds of attaining TB treatment success compared to those who did not have access to social protection interventions.”*

3. We thank Reviewer 1 for their attention to detail, and agree that the manuscript’s stated objective to report on both TB treatment and socioeconomic outcomes are not aligned with the results from the study. We have added the following sentence to the main text (page 17, lines 428-430) to clarify this discrepancy:

“*Although we aimed to provide a comprehensive assessment of the impact of social protection on socioeconomic outcomes, our outcomes of interest were underrepresented and insufficient to synthesize*.”

4. We appreciate Reviewer 2’s thoughtful comment about providing an overview about what conducting a systematic review entails. We therefore added the following to our methods section (page 5, line 125-131):

*“Our systematic review and meta-analysis were conducted in accordance with PRISMA guidelines. Specifically, we 1) literately developed a list of search terms and a search strategy, 2) searched for relevant articles in a pre-determined number of databases, 3) systematically screened titles and abstracts for potentially eligible articles, 4) read the full text of articles deemed eligible for full text review, 5) agreed upon articles that met criteria for inclusion, 6) extracted relevant data from each study, 7) conducted quality and risk of bias assessments, and 8) synthesized available evidence.”*

5. We appreciate Reviewer 1’s observation about missing and non-extractable data. We have updated our submission to include the granular risk of bias assessments, which downgrade study quality for missing data (S2, Tables 5 and 6). We have also added S2, Table 7, which summarizes which studies reported on TB treatment success but had non-extractable data for inclusion in the meta-analysis. Additionally, we clarified where and how missing data affected quality assessments (page 11, lines 274-280).
6. We agree with Reviewer 1’s thoughtful comment regarding implementation outcomes, and that the discussion regarding synthesis of these outcomes should be expanded. We added the following sentence to page 11, line 271-273:

*“Fidelity, coverage, or feasibility of social protection interventions was not consistently reported, which precluded analysis of implementation outcomes.”*

We also expanded our discussion section to describe how implementation of social protection in different contexts likely contributes to heterogeneity (page 17, lines 392-398):

*“Our subgroup analyses revealed two sources of between-study heterogeneity. First, WHO region proved to be a significant source of between-study heterogeneity. These findings suggest that the magnitude of the effect of social protection can vary substantially given the sociodemographic features of a population, baseline TB epidemiology, and effective implementation of social protection interventions. Second, whether an intervention was TB specific or TB sensitive was also a significant source of between-study heterogeneity, with TB-specific programs conferring a much greater effect on TB treatment success.”*

7. Both Reviewer 1 and Reviewer 2 raised an important concern that our manuscript does not provide adequate policy recommendations. We have added to our discussion to provide more detail about actionable guidance (page 16, lines 398-406), particularly pertaining to TB sensitive versus specific social protection interventions:

*“These findings parallel those by Lima Ferriera et al.^54^, and are perhaps the most significant in terms of health and social welfare policy. Albeit moderate, TB-sensitive social protection interventions still significantly improve the odds of TB treatment success, despite not being targeted specifically towards individuals with TB. Countries with existing, robust TB-sensitive interventions may consider interventions to ensure that TB-affected individuals have access to existing social protection interventions. TB specific interventions, which may be less costly, can confer a much greater benefit. National TB programs should consider incorporating social protection interventions to ensure higher rates of TB treatment success.”*

8. We thank Reviewer 2 for their observation that the analysis of socioeconomic outcomes was underdeveloped in the results section. We have added additional description of each study in the results section (page 10, lines 258-263), as well as additional commentary in the discussion (pages 17-18, 432-434).

Results: “*Wingfield et al., for example, found that a conditional cash transfer defrayed 20% of total costs, 39% of direct costs, and 19% of lost income. Among poorer households and female participants, the cash transfer defrayed total costs 22% and 23%, respectively.^3^ Similarly, Florentino et al. found that social protection reduced catastrophic costs to a much greater extent amount individuals with drug-resistant TB, who incurred higher costs than individuals with drug-susceptible TB.^32^”*

Discussion: “*Further, such findings would be key to developing person-and household centered approaches to developing and implementing social protection interventions to improve socioeconomic outcomes.”*

9. To address Reviewer 1’s concern regarding bias, we note that we did use GRADE as recommended by the reviewer (S4, Table 1). In the main text (page 11, lines 278-280), we state:

*“Assessments of certainty and the quality of evidence of across studies were evaluated using the Grading of Recommendations, Assessment, Development, and Evaluation (GRADE) (further described in* ***S4, Table 1****).”*

10. We appreciate Reviewer 2’s thoughtful comment that we should engage more fully with prior literature to better position the novelty and contribution of the present study. We have subsequently added additional detail to the introduction (page 5, lines 109-117)

*“While prior studies, including some systematic reviews, have found that social protection interventions can improve TB treatment outcomes,^11^ they were limited in their ability to widely inform policy and programmatic implementation of various types of social protections in a wide array of settings. De Andrade et al, for example, limited their meta-analysis to randomized control trials.^12^ While high quality, these results exclude a substantial body of evidence generated by high quality observational studies on a range of TB treatment outcomes. Another study conducted by Richterman et al. focused only on cash transfers, thereby not including studies that describe other types of social protection interventions such as food assistance or psychosocial support.^11^”*

We again thank the reviewers and editors for their thoughtful, detailed, insightful responses. We greatly appreciate the opportunity to revise our manuscript.
