# Supplementary material for: The impact of social protection interventions on treatment and socioeconomic outcomes of tuberculosis-affected people and households in low income, high burden settings: A systematic review and meta-analysis
Source: PLOS Glob Public Health. 2026 Sep 3;6(9):e0006704. doi: 10.1371/journal.pgph.0006704 (PMC13541142; doi:10.1371/journal.pgph.0006704)
Supplement: Peer review history

# Author comment: Author response to Decision Letter 0

**DOI:** 10.1371/journal.pgph.0006704.r001

31 Mar 2026

Attachment Submitted filename: PLOS GPH Response to reviewers March.docx

# Peer review: Decision Letter 0

**DOI:** 10.1371/journal.pgph.0006704.r002

**Authors:** Graeme Hoddinott

23 Apr 2026

PGPH-D-26-01201

The impact of social protection interventions on treatment and socioeconomic outcomes of tuberculosis-affected people and households in low income, high burden settings: A systematic review and meta-analysis

PLOS Global Public Health

Dear Dr. Hudson,

Thank you for submitting your manuscript to PLOS Global Public Health. After careful consideration, we feel that it has merit but does not fully meet PLOS Global Public Health’s publication criteria as it currently stands. Therefore, we invite you to submit a revised version of the manuscript that addresses the points raised during the review process.

==============================

EDITOR:

The reviewers have raised a number of concerns / issues to address with which I agree. Please take the time to consider these carefully. Also, note that both also congratulate you on an important manuscript and by-and-large sound approach. The required revisions are about being a bit more circumspect and critical.

==============================

Please submit your revised manuscript by Jun 07 2026 11:59PM. If you will need more time than this to complete your revisions, please reply to this message or contact the journal office at globalpubhealth@plos.org. When you're ready to submit your revision, log on to https://www.editorialmanager.com/pgph/ and select the 'Submissions Needing Revision' folder to locate your manuscript file.

Please include the following items when submitting your revised manuscript:

- A letter that responds to each point raised by the editor and reviewer(s). You should upload this letter as a separate file labeled 'Response to Reviewers'.
- A marked-up copy of your manuscript that highlights changes made to the original version. You should upload this as a separate file labeled 'Revised Manuscript with Track Changes'.
- An unmarked version of your revised paper without tracked changes. You should upload this as a separate file labeled 'Manuscript'.

Guidelines for resubmitting your figure files are available below the reviewer comments at the end of this letter.

As the corresponding author, your ORCID iD is verified in the submission system and will appear in the published article. PLOS supports the use of ORCID, and we encourage all coauthors to register for an ORCID iD and use it as well. Please encourage your coauthors to verify their ORCID iD within the submission system before final acceptance, as unverified ORCID iDs will not appear in the published article. *Only*  the individual author can complete the verification step; PLOS staff *cannot*  verify ORCID iDs on behalf of authors.

We look forward to receiving your revised manuscript.

Kind regards,

Graeme Hoddinott, Ph.D

Academic Editor

PLOS Global Public Health

Journal Requirements:

1. As required by our policy on Data Availability, please ensure your manuscript or supplementary information includes the following:

A numbered table of all studies identified in the literature search, including those that were excluded from the analyses.

For every excluded study, the table should list the reason(s) for exclusion.

If any of the included studies are unpublished, include a link (URL) to the primary source or detailed information about how the content can be accessed.

A table of all data extracted from the primary research sources for the systematic review and/or meta-analysis. The table must include the following information for each study:

Name of data extractors and date of data extraction

Confirmation that the study was eligible to be included in the review.

All data extracted from each study for the reported systematic review and/or meta-analysis that would be needed to replicate your analyses.

If data or supporting information were obtained from another source (e.g. correspondence with the author of the original research article), please provide the source of data and dates on which the data/information were obtained by your research group.

If applicable for your analysis, a table showing the completed risk of bias and quality/certainty assessments for each study or outcome.  Please ensure this is provided for each domain or parameter assessed. For example, if you used the Cochrane risk-of-bias tool for randomized trials, provide answers to each of the signalling questions for each study. If you used GRADE to assess certainty of evidence, provide judgements about each of the quality of evidence factor. This should be provided for each outcome.

An explanation of how missing data were handled.

This information can be included in the main text, supplementary information, or relevant data repository. Please note that providing these underlying data is a requirement for publication in this journal, and if these data are not provided your manuscript might be rejected.

2. Please provide a detailed online Financial Disclosure statement. This is published with the article. It must therefore be completed in full sentences and contain the exact wording you wish to be published.

a) State the initials, alongside each funding source, of each author to receive each grant. For example: “This work was supported by the National Institutes of Health (####### to AM; ###### to CJ) and the National Science Foundation (###### to AM).”

b) State what role the funders took in the study. If the funders had no role in your study, please state: “The funders had no role in study design, data collection and analysis, decision to publish, or preparation of the manuscript.”

For more information, please go to our submission guidelines:

https://journals.plos.org/globalpublichealth/s/submission-guidelines#loc-financial-disclosure-statement

3. Please ensure that the funders and grant numbers match between the Financial Disclosure field and the Funding Information tab in your submission form. Note that the funders must be provided in the same order in both places as well.

4. Please update your online Competing Interests statement. If you have no competing interests to declare, please state: "The authors have declared that no competing interests exist."

5. Please provide separate main figure files in .tif or .eps format only and remove any figures embedded in your manuscript file. Please also ensure that all files are under our size limit of 10MB. Please leave the figure captions in the manuscript.

For more information about how to convert your figure files please see our guidelines: https://journals.plos.org/globalpublichealth/s/figures

6. We notice that your supplementary materials are included in the manuscript file. Please remove them and upload them with the file type 'Supporting Information'. Please ensure that each Supporting Information file has a legend listed in the manuscript before or after the references list.

7. We have noticed that you have uploaded Supporting Information files, but you have not included a list of legends. Please add a full list of legends for your Supporting Information files before or after the references list.

8. If the reviewer comments include a recommendation to cite specific previously published works, please review and evaluate these publications to determine whether they are relevant and should be cited. There is no requirement to cite these works unless the editor has indicated otherwise.

Additional Editor Comments (if provided):

[Note: HTML markup is below. Please do not edit.]

Reviewers' comments:

Reviewer's Responses to Questions

**Comments to the Author**

1. Does this manuscript meet PLOS Global Public Health’s publication criteria?

Reviewer #1: Yes

Reviewer #2: Yes

**********

2. Has the statistical analysis been performed appropriately and rigorously?

Reviewer #1: Yes

Reviewer #2: Yes

**********

3. Have the authors made all data underlying the findings in their manuscript fully available (please refer to the Data Availability Statement at the start of the manuscript PDF file)?

The PLOS Data policy

Reviewer #1: Yes

Reviewer #2: Yes

**********

4. Is the manuscript presented in an intelligible fashion and written in standard English?

Reviewer #1: Yes

Reviewer #2: Yes

**********

Reviewer #1: This is a well-conducted and policy-relevant systematic review with important implications for TB control. However, substantial revisions are required to strengthen the interpretation of findings, address heterogeneity, and improve balance between outcomes before the manuscript is suitable for publication:

1) There is a very high heterogeneity (I²=94.5%) reported but it is insufficiently explored or interpreted. Authors should provide deeper investigation (subgroup analyses, sensitivity analyses) and clearer discussion of implications.

2) The conclusion that social protection “significantly improves” outcomes may be overstated given heterogeneity and predominance of observational studies. Authors are advised to give a more cautious and tempered down interpretation.

3) The manuscript is heavily skewed toward treatment outcomes, while socioeconomic outcomes are underrepresented and insufficiently synthesized. Authors should align with the stated objectives.

4) Key dimensions (coverage, fidelity, feasibility) are poorly reported and not systematically analyzed. Authors should expand discussion and highlight this gap more critically.

5) Variability in definitions of “social protection” and socioeconomic outcomes is acknowledged but not adequately operationalized. Authors should consider categorization or stricter inclusion criteria.

6) Could authors provide more details on how missing or non-extractable data was handled, how they decided which studies would be excluded from the meta-analysis, and the rationale for the meta-regression variable?

7) Although tools are applied, the implications of bias on pooled estimates are not critically discussed. could authors consider GRADE or clearer certainty of evidence framing?

8) Overrepresentation of middle-income settings (e.g. Brazil) limits applicability to low-income, high-burden African contexts. This should be more explicitly discussed and problematized

9) The introduction and discussion contain repetitive arguments regarding social protection importance. Please revise.

10) While policy relevance is mentioned, recommendations remain generic. Authors should provide more concrete, actionable guidance for TB programs.

Reviewer #2: First, I would like to congratulate the authors for conducting a study on a highly timely and policy-relevant topic for global TB elimination, particularly in light of the increasing emphasis on social protection within strategies to end tuberculosis as a public health problem. The manuscript addresses an important and underexplored area and is based on a rigorous review process. I offer the comments below with the aim of clarifying some issues identified during reading and further strengthening the manuscript.

In the Introduction, in the final paragraph, the manuscript refers to “systematic reviews” in the plural, although only one prior review is mentioned. Moreover, to the best of my knowledge, the existing body of evidence already includes previous systematic reviews, scoping reviews, and meta-analyses on the effects of TB-specific and TB-sensitive interventions implemented during TB treatment to provide social protection for people affected by TB. I would encourage the authors to engage more fully with this prior literature in order to better position the novelty and contribution of the present study, and to avoid unintentionally minimizing the state of the art already established on this topic.

In the Methods section, I would also like to commend the authors for the methodological rigor of the review. The protocol registration, database coverage, and use of established risk-of-bias tools are all important strengths. However, I felt that the section would benefit from a short introductory paragraph clearly outlining the main stages of the systematic review and meta-analysis, especially for readers who may be less familiar with evidence synthesis methods. In addition, the decision to adopt the World Bank definition of social protection is valuable in terms of conceptual standardization, but it also introduces an important challenge: this definition brings together interventions of very different natures, including cash transfers, nutritional support, and psychosocial support. This degree of heterogeneity may affect the comparability of included studies and, consequently, the interpretation of the pooled estimates. I would encourage the authors to acknowledge this more explicitly, either in the Methods or in the Limitations.

In the Results, one of the first points that draws attention, already from the abstract, is the very high heterogeneity reported in the meta-analysis (I² = 94.5%). While the use of a random-effects model is appropriate, I believe this degree of heterogeneity should be given greater prominence and interpreted more critically throughout the manuscript. In its current form, the statement that social protection interventions “significantly improve odds” of treatment success is statistically correct, but the magnitude and consistency of the pooled effect should be interpreted with caution given the substantial between-study variability. This issue deserves more explicit discussion, particularly in relation to whether the pooled estimate can reasonably be interpreted as a single overall effect across such diverse interventions and settings.

Still in the Results, I found the analysis of socioeconomic outcomes somewhat underdeveloped. Although the number of studies reporting these outcomes was smaller and the measures were highly variable, this is one of the most innovative and policy-relevant dimensions of the review. The manuscript notes that only a limited number of studies assessed outcomes such as catastrophic costs and dissaving, but the narrative synthesis could be more analytically developed, especially because these outcomes are central to moving beyond a strictly biomedical understanding of TB and toward a person- and household-centered approach.

In addition, the distinction between TB-specific and TB-sensitive social protection interventions is clearly introduced in the Methods, but this categorization is not explored comparatively in the Results or Discussion. I believe the manuscript would benefit from a more explicit analysis, since this distinction has important implications for policy design, implementation feasibility, and targeting in high-burden settings.

Finally, in the Conclusion, and also in the section “How this study might affect research, practice or policy,” I felt there was room to strengthen the practical implications of the findings for the contexts highlighted by the study itself, namely low-income and high-burden settings. At present, the manuscript concludes that social protection improves treatment outcomes and may inform future implementation, but it says relatively little about what these findings imply for the design of future social protection programmes, how different intervention types might be prioritized, or what policymakers in high-burden settings can realistically take from the available evidence. A more explicit reflection on the operational and policy relevance of the findings would make the paper more useful for both researchers and decision-makers.

**********

what does this mean?). If published, this will include your full peer review and any attached files.

**Do you want your identity to be public for this peer review?**  If you choose “no”, your identity will remain anonymous but your review may still be made public.

For information about this choice, including consent withdrawal, please see our Privacy Policy

Reviewer #1: No

Reviewer #2: **Yes:** Melisane Regina Lima Ferreira

**********

[NOTE: If reviewer comments were submitted as an attachment file, they will be attached to this email and accessible via the submission site. Please log into your account, locate the manuscript record, and check for the action link "View Attachments". If this link does not appear, there are no attachment files.]

# Author comment: Author response to Decision Letter 1

**DOI:** 10.1371/journal.pgph.0006704.r003

4 Jun 2026

Attachment Submitted filename: Responses to PLOS GPH 4 June 2026.docx

# Peer review: Decision Letter 1

**DOI:** 10.1371/journal.pgph.0006704.r004

**Authors:** Graeme Hoddinott

9 Jul 2026

PGPH-D-26-01201R1

The impact of social protection interventions on treatment and socioeconomic outcomes of tuberculosis-affected people and households in low income, high burden settings: A systematic review and meta-analysis

PLOS Global Public Health

Dear Dr. Hudson,

Thank you for submitting your manuscript to PLOS Global Public Health. After careful consideration, we feel that it has merit but does not fully meet PLOS Global Public Health’s publication criteria as it currently stands. Therefore, we invite you to submit a revised version of the manuscript that addresses the points raised during the review process.

==============================

EDITOR:

As suggested by reviewers, just a few minor changes requested.

==============================

Please submit your revised manuscript by Aug 08 2026 11:59PM. If you will need more time than this to complete your revisions, please reply to this message or contact the journal office at globalpubhealth@plos.org. When you're ready to submit your revision, log on to https://www.editorialmanager.com/pgph/ and select the 'Submissions Needing Revision' folder to locate your manuscript file.

Please include the following items when submitting your revised manuscript:

- A letter that responds to each point raised by the editor and reviewer(s). You should upload this letter as a separate file labeled 'Response to Reviewers'.
- A marked-up copy of your manuscript that highlights changes made to the original version. You should upload this as a separate file labeled 'Revised Manuscript with Track Changes'.
- An unmarked version of your revised paper without tracked changes. You should upload this as a separate file labeled 'Manuscript'.

Guidelines for resubmitting your figure files are available below the reviewer comments at the end of this letter.

As the corresponding author, your ORCID iD is verified in the submission system and will appear in the published article. PLOS supports the use of ORCID, and we encourage all coauthors to register for an ORCID iD and use it as well. Please encourage your coauthors to verify their ORCID iD within the submission system before final acceptance, as unverified ORCID iDs will not appear in the published article. *Only*  the individual author can complete the verification step; PLOS staff *cannot*  verify ORCID iDs on behalf of authors.

We look forward to receiving your revised manuscript.

Kind regards,

Graeme Hoddinott, Ph.D

Academic Editor

PLOS Global Public Health

**Journal Requirements:**

If the reviewer comments include a recommendation to cite specific previously published works, please review and evaluate these publications to determine whether they are relevant and should be cited. There is no requirement to cite these works unless the editor has indicated otherwise.

Please review your reference list to ensure that it is complete and correct. If you have cited papers that have been retracted, please include the rationale for doing so in the manuscript text, or remove these references and replace them with relevant current references. Any changes to the reference list should be mentioned in the rebuttal letter that accompanies your revised manuscript. If you need to cite a retracted article, indicate the article’s retracted status in the References list and also include a citation and full reference for the retraction notice.

**Additional Editor Comments (if provided):**

[Note: HTML markup is below. Please do not edit.]

Reviewers' comments:

Reviewer's Responses to Questions

**Comments to the Author**

Reviewer #1: All comments have been addressed

Reviewer #2: All comments have been addressed

**********

publication criteria?

Reviewer #1: Yes

Reviewer #2: Yes

**********

3. Has the statistical analysis been performed appropriately and rigorously?

Reviewer #1: Yes

Reviewer #2: Yes

**********

4. Have the authors made all data underlying the findings in their manuscript fully available (please refer to the Data Availability Statement at the start of the manuscript PDF file)?

The PLOS Data policy

Reviewer #1: Yes

Reviewer #2: Yes

**********

5. Is the manuscript presented in an intelligible fashion and written in standard English?

Reviewer #1: Yes

Reviewer #2: Yes

**********

**Reviewer #1:**  The authors have addressed all major concerns adequately. There are only minor polish ups left, before acceptance for publication:

1) Some grammatical errors need to be corrected: social protection inventions should be interventions; were generally had high levels should be generally had high levels; amount individuals should be among individuals; statically significant should be statistically significant; literately developed should be iteratively developed. Please do a final language proofreading and editing.

2) The abstract reports 26 studies in the meta-analysis, but the results section reports 24 studies and then later in the meta-regression you report 26; please authors correct this and be consistent.

3) the wording " can dramatically improve TB treatment outcomes" is an overstatement; it is best replaced by "are associated with substantially improved treatment outcomes".

4) in the discussion briefly mention residual confounding in observational studies, publication bias if funnel plot/egger's test was assessed and finally why prediction intervals are more informative than pooled ORs when heterogeneity is high.

**Reviewer #2:**  Thank you to the authors for the careful revision. The manuscript has improved substantially, particularly through the more explicit discussion of heterogeneity. I also particularly appreciate the subgroup comparison between TB-specific and TB-sensitive interventions, which adds important policy relevance to the study. Overall, I believe the main substantive concerns have been adequately addressed.

I only have a few minor comments for final revision:

In the Methods, the sentence stating that "studies describing type of intervention were not included" (line 175) is unclear and seems inconsistent with the rest of the manuscript, since intervention types were included and analyzed later. This likely needs rewording.

In the Results, "intervention type" (line 337) is listed twice in the description of subgroup analyses.

There is an inconsistency in the number of studies included in the meta-analysis: the abstract and Table 2 indicate 26 studies, while the Results section states 24 studies. Please check and correct this throughout the manuscript.

The citation "Lima Ferriera et al." should be corrected to "Ferreira et al.".

Congratulations!

**********

what does this mean?). If published, this will include your full peer review and any attached files.

**Do you want your identity to be public for this peer review?**  If you choose “no”, your identity will remain anonymous but your review may still be made public.

For information about this choice, including consent withdrawal, please see our Privacy Policy

Reviewer #1: No

Reviewer #2: No

**********

[NOTE: If reviewer comments were submitted as an attachment file, they will be attached to this email and accessible via the submission site. Please log into your account, locate the manuscript record, and check for the action link "View Attachments". If this link does not appear, there are no attachment files.]

**Figure Resubmissions:**

# Author comment: Author response to Decision Letter 2

**DOI:** 10.1371/journal.pgph.0006704.r005

28 Jul 2026

Attachment Submitted filename: V2 Plos response to reviewers 28 July 2026.pdf

# Peer review: Decision Letter 2

**DOI:** 10.1371/journal.pgph.0006704.r006

**Authors:** Graeme Hoddinott

30 Jul 2026

The impact of social protection interventions on treatment and socioeconomic outcomes of tuberculosis-affected people and households in low income, high burden settings: A systematic review and meta-analysis

PGPH-D-26-01201R2

Dear Dr. Hudson,

We are pleased to inform you that your manuscript 'The impact of social protection interventions on treatment and socioeconomic outcomes of tuberculosis-affected people and households in low income, high burden settings: A systematic review and meta-analysis' has been provisionally accepted for publication in PLOS Global Public Health.

Before your manuscript can be formally accepted you will need to complete some formatting changes, which you will receive in a follow up email. A member of our team will be in touch with a set of requests.

Please note that your manuscript will not be scheduled for publication until you have made the required changes, so a swift response is appreciated.

IMPORTANT: The editorial review process is now complete. PLOS will only permit corrections to spelling, formatting or significant scientific errors from this point onwards. Requests for major changes, or any which affect the scientific understanding of your work, will cause delays to the publication date of your manuscript.

If your institution or institutions have a press office, please notify them about your upcoming paper to help maximize its impact. If they'll be preparing press materials, please inform our press team as soon as possible -- no later than 48 hours after receiving the formal acceptance. Your manuscript will remain under strict press embargo until 2 pm Eastern Time on the date of publication. For more information, please contact globalpubhealth@plos.org.

Thank you again for supporting Open Access publishing; we are looking forward to publishing your work in PLOS Global Public Health.

Best regards,

Graeme Hoddinott, Ph.D

Academic Editor

PLOS Global Public Health

***********************************************************

Reviewer Comments (if any, and for reference):

Reviewer's Responses to Questions

**Comments to the Author**

Reviewer #1: All comments have been addressed

Reviewer #2: All comments have been addressed

**********

publication criteria?

Reviewer #1: Yes

Reviewer #2: Yes

**********

3. Has the statistical analysis been performed appropriately and rigorously?

Reviewer #1: Yes

Reviewer #2: Yes

**********

4. Have the authors made all data underlying the findings in their manuscript fully available (please refer to the Data Availability Statement at the start of the manuscript PDF file)?

The PLOS Data policy

Reviewer #1: Yes

Reviewer #2: Yes

**********

5. Is the manuscript presented in an intelligible fashion and written in standard English?

Reviewer #1: Yes

Reviewer #2: Yes

**********

Reviewer #1: No further comments. Authors have addressed all concerns adequately

Reviewer #2: (No Response)

**********

what does this mean?). If published, this will include your full peer review and any attached files.

**Do you want your identity to be public for this peer review?**  If you choose “no”, your identity will remain anonymous but your review may still be made public.

For information about this choice, including consent withdrawal, please see our Privacy Policy

Reviewer #1: No

Reviewer #2: **Yes:** Melisane Regina Lima Ferreira

**********
